# Siderophore production by the lichen fungus *Xanthoria parietina* supports its algal symbiont

**DOI:** 10.1038/s41467-026-74988-9

**Published:** 2026-07-02

**Authors:** Isidor Happacher, Gregor Pichler, Beate Abt, Gulnara Tagirdzhanova, Simon Oberegger, Martin Kraihammer, Klaus Faserl, Bettina Sarg, Clemens Decristoforo, Roman Türk, Günther Winkelmann, Nicholas J. Talbot, Matthias Brock, Ilse Kranner, Kurt Haselwandter, Hubertus Haas

**Affiliations:** 1 Institute of Molecular Biology, Biocenter, Medical University Innsbruck, Innrain 80-82, Innsbruck, Austria; 2 Department of Botany, University of Innsbruck, Sternwartestraße 15, Innsbruck, Austria; 3 HBLFA für Gartenbau und Österreichische Bundesgärten, Federal Ministry of Agriculture and Forestry, Climate and Environmental Protection, Regions and Water Management, of the Republic of Austria, Grünbergstraße 24, Vienna, Austria; 4 The Sainsbury Laboratory, University of East Anglia, Norwich Research Park, Colney Lane, Norwich, NR47UH United Kingdom; 5 Department of Ecology, Environment and Plant Sciences, Stockholm University, Stockholm, Sweden; 6 Department of Nuclear Medicine, Medical University Innsbruck, Anichstrasse 35, Innsbruck, Austria; 7 Protein Core Facility, Institute of Medical Biochemistry, Biocenter, Medical University Innsbruck, Innrain 80-82, Innsbruck, Austria; 8 Department of Environment & Biodiversity, Paris Lodron University Salzburg, Hellbrunnerstraße 34, Salzburg, Austria; 9 Department of Microbiology & Biotechnology, University of Tübingen, Auf der Morgenstelle 28, Tübingen, Germany; 10 Fungal Biology Group, School of Life Sciences, University of Nottingham, University Park, Nottingham United Kingdom; 11 Department of Microbiology, University of Innsbruck, Technikerstrasse 25, Innsbruck, Austria

**Keywords:** Fungal biology, Fungi, Symbiosis

## Abstract

Lichens are symbiotic associations between a fungal mycobiont and a photosynthetic photobiont. They thrive in nutrient-poor environments; yet the mechanisms underlying their adaptation to iron limitation remained largely unknown. Here, we characterize the iron acquisition system of *Xanthoria parietina*, a globally distributed lichen-forming fungus associated with the microalgal photobiont *Trebouxia decolorans*. We demonstrate that the mycobiont produces the siderophore ferrichrome and possesses the full genetic repertoire not only for siderophore biosynthesis, but also reductive iron assimilation, iron detoxification, and regulation. The ferrichrome-synthesizing non-ribosomal peptides synthetase exhibits a lichen-specific compact architecture but retains functionality when heterologously expressed in a non-lichenized ascomycete. Transcriptomic analysis and ferrichrome quantification reveal substrate-dependent regulation of the siderophore system. Importantly, ferrichrome promotes photobiont growth independent of extracellular iron reduction, indicating direct utilization. These findings provide the functional evidence of siderophore-mediated iron acquisition in a lichen symbiosis and highlight ferrichrome as a key mediator of mutualistic nutrient exchange.

## Abstract (web summary)

The mechanisms underlying the adaptation of lichens to nutrient-poor environments are poorly understood. Here, Happacher et al. show that a globally distributed lichen fungus produces an iron-scavenging molecule that promotes growth of its algal partner.

## Introduction

The term “symbiosis” was originally coined to describe the mutualistic relationship of lichens, discovered in 1869^1^. Today, lichens are defined as self-sustaining ecosystems formed by a predominant fungus (the mycobiont), which builds the majority of the lichen biomass, in association with extracellular green algal and/or cyanobacterial photosynthetic partners (called photobionts) and an indeterminate number of other microorganisms, including bacteria and other fungi^2^. The photobiont produces carbohydrates through photosynthesis, while the mycobiont provides structure, a protective environment and nutrient supply for the photobiont^3^. Lichens are characterized by their ability to colonize extreme habitats that are limited in nutrients and subject to many other stress factors, such as solar radiation, salt stress and significant variations in hydration and desiccation^4^. In extreme environments, lichens are an important food source for animals such as reindeer in northern hemisphere during the winter. However, our understanding of the physiological and biochemical processes underlying lichen symbiosis, and of how lichens cope with such harsh conditions, is very limited.

Virtually all life depends on iron supply for a wide range of biological processes. Despite being the fourth most abundant element in the Earth’s crust^5^, iron is only bioavailable in limited quantities in aerobic niches due to its tendency to oxidize into hardly soluble compounds such as ferric hydroxides^6^. As in other organisms, iron is expected to play a central role in the physiology of lichens. As the photobiont partner is embedded within the hyphal mycobiont cortex, iron must be supplied by the mycobiont by as yet unknown mechanisms. In general, fungi have evolved high-affinity iron acquisition systems such as reductive iron assimilation (RIA) and siderophore-mediated iron acquisition (SIA)^7^. Siderophores are low-molecular-mass (mostly <1 kDa), ferric iron-specific chelators utilized by bacteria and fungi for iron acquisition, and in the case of fungi, also for intracellular iron transport and storage^7^. The majority of fungal species belonging to *Ascomycota* and *Basidiomycota* synthesize hydroxamate-class siderophores containing three hydroxamate groups based on the basic building block *N*^*5*^*-*acyl-*N*^*5*^-hydroxy-l-ornithine^8^. Fungal hydroxamate-class siderophores are classified into structurally distinct types: fusarinines, coprogens, ferrichromes, and rhodotorulic acid^8^. Moreover, linear trishydroxamates (basidiochrome, basidioferrin and coprinoferrin) have been identified in *Basidiomyceta*^9–11^. The molecular basis of fungal SIA has been most thoroughly studied in *Aspergillus fumigatus*. This mold species produces two fusarinine-type siderophores termed triacetylfusarinine C (TAFC) and fusarinine C, and two ferrichrome-type siderophores, ferricrocin and hydroxyferricrocin^7^. TAFC, fusarinine C and ferricrocin are secreted for uptake of environmental iron; ferricrocin is also used for intracellular transport and storage of iron within hyphae and hydroxyferricrocin for conidial iron storage^7,12^. The siderophore biosynthetic pathway involving ornithine monooxygenase (SidA), mevalonyl-coenzyme A (CoA) ligase (SidI), mevalonyl-CoA hydratase (SidH), transacylases (SidF, SidG and SidL), and non-ribosomal peptide synthetases (NRPSs; SidC and SidD)^7,13,14^ is shown in Supplementary Fig. S1. After extracellular iron chelation, the ferrichrome-iron chelates are taken up by specific siderophore-iron transporters (SITs), MirD for fusarinine C, MirB for TAFC, Sit1 and Sit2 for coprogen- and ferrichrome-type siderophores, with Sit1 also mediating utilization of bacterial ferrioxamine-type siderophores^15–17^. The intracellular utilization of iron chelated by TAFC and fusarinine C involves siderophore hydrolysis mediated by the esterases SidJ and EstB^18^.

In RIA, ferric iron is reduced by membrane-localized metalloreductases, such as *A. fumigatus* FreB and subsequently re-oxidized and taken up by a protein complex consisting of a ferroxidase and an iron permease, termed FetC and FtrA in *A. fumigatus*^7^. Iron homeostasis is controlled by the two iron-sensing transcription factors SreA and HapX, with HapX cooperating with the CCAAT-binding complex^7^. SreA prevent excessive iron uptake by repressing SIA and RIA^19^. HapX represses iron-consuming pathways to spare iron under conditions of iron limitation, and activates iron detoxification via vacuolar deposition by the CccA transporter under conditions of iron excess^7,20,21^. Siderophore biosynthesis, TAFC uptake, and HapX-mediated iron regulation are crucial for virulence of *A. fumigatus* in murine and invertebrate infection models^7,17,22^. *Mucoromycota* species appear to lack hydroxamate-class siderophores but use carboxylate-class siderophores such as rhizoferrin, originally isolated from *Rhizopus microsporus*^23^.

Substrate assembly of nonribosomal peptides by NRPSs proceeds by repeated cycles catalyzed by three core domains, adenylation (A), thiolation (T or peptidyl carrier protein) and condensation (C) domains, which together form a minimal NRPS module^24^. A-domains ATP-activate substrate carboxylate groups and transfer them to the adjacent T-domain, thereby determining substrate selection; C-domains then catalyze peptide or ester bond formation between substrates tethered to the T-domains of adjacent modules. Chain release occurs via a thioesterase or, more commonly in fungi, through a terminal C-domain (C_T_) mediating intramolecular cyclisation^25^. Ferrichrome-type siderophores, cyclic hexapeptides, are synthesized by NRPSs with nonlinear domain organizations involving iterative domain use^26^. For example, the ferricrocin-synthesizing NRPS SidC from *Aspergillus nidulans* and its *A. fumigatus* ortholog iteratively assemble three distinct precursor types, three *N*^*5*^-acetyl-*N*^*5*^-hydroxy- l-ornithine moieties, two glycine and one serine residues^27,28^, consistent with its three A-domains. Ferrichrome assembly, in contrast, requires only two precursor types, three *N*^*5*^-acetyl-*N*^*5*^-hydroxy-l-ornithine moieties and three glycine residues; so an optimized NRPS would be expected to contain only two A-domains. Nevertheless, the ferrichrome NRPS Sid2 from *Ustilago maydis* contains three A-domains^29^, while Sib1 from *Schizosaccharomyces pombe* contains two complete and one degenerate A-domain along with multiple C-domains^30^.

Mutualistic fungi including ericoid (e.g., *Hyaloscypha hepaticicola* and *Oidiodendron griseum*), orchidaceous (e.g., *Ceratobasidium* spp. and *Rhizoctonia* spp.) and ectomycorrhizal (e.g., *Cenococcum geophilum, Laccaria bicolor and Laccaria laccata*) fungal species also employ hydroxamate-class siderophores^31,32^. Root tissues of plants colonized by different species of arbuscular mycorrhizal fungi were found to contain glomuferrin, a dehydration product of rhizoferrin^23^. Moreover, the perennial ryegrass (*Lolium perenne*) endophyte *Epichloë festucae* employs the atypical ferrichrome-type siderophore epichloënin A as extracellular siderophore and ferricrocin as intracellular siderophore^33^. SIA and RIA are complementary systems required to maintain the mutualistic relationship between *E. festucae* and perennial ryegrass^33^. In summary, siderophores are produced by fungal partners of mycorrhizal and endophytic associations and may play a role in symbiosis.

However, little is known about the role of iron homeostatic mechanisms in lichens. Numerous mycobiont and photobiont pathways, such as oxidative phosphorylation, the TCA cycle, oxidative stress detoxification, DNA replication as well as the biosynthesis of amino acids, nucleotides, and sterols, depend on iron or iron-containing cofactors including iron-sulfur clusters, heme and siroheme. Iron dependence of the photobiont is even higher, because the photosynthetic electron transport chain is estimated to account for more than 50% of cellular iron in microalgae^34^.

By analogy with other mutualistic fungi, we hypothesized that the lichen-forming mycobiont *Xanthoria parietina*, a globally distributed species within the *Lecanoromycetes* class/*Ascomycota* phylum^35^, synthesizes siderophores to acquire iron not only for its own metabolic needs but also to support its photobiont, the green microalga *Trebouxia decolorans*. Compared to many other lichen species, *X. parietina* can survive in agricultural, urbanized and industrialized regions, resisting environmental pollution^36,37^ and is commonly found on rocks and tree bark^38,39^. For the *X. parietina* mycobiont, genomic and transcriptomic resources are available facilitating molecular interpretation. In addition, axenic cultures of both symbionts are available, enabling the production of sufficient biomass for biochemical and physiological analyses - an advantage that is uncommon among lichen mycobionts.

In this study, we show that the *X. parietina* mycobiont synthesizes ferrichrome both in lichen thalli and in axenic culture. Genome mining has identified the genetic basis of its SIA system, as well as its potential for RIA and iron detoxification by vacuolar deposition. A ferrichrome-synthesizing NRPS, validated by heterologous expression in *A. fumigatus*, features a unique compact domain architecture that is highly conserved in other lichen mycobiont species. The siderophore system was transcriptionally up-regulated in lichen thalli grown on concrete compared to organic substrates and ferrichrome was found to promote the growth of the *X. parietina* photobiont *T. decolorans*.

## Results

### *A. fumigatus* bioassays indicate the presence of siderophores in *X. parietina* lichen thallus and axenic mycobiont culture supernatant

**Fig. 1:**
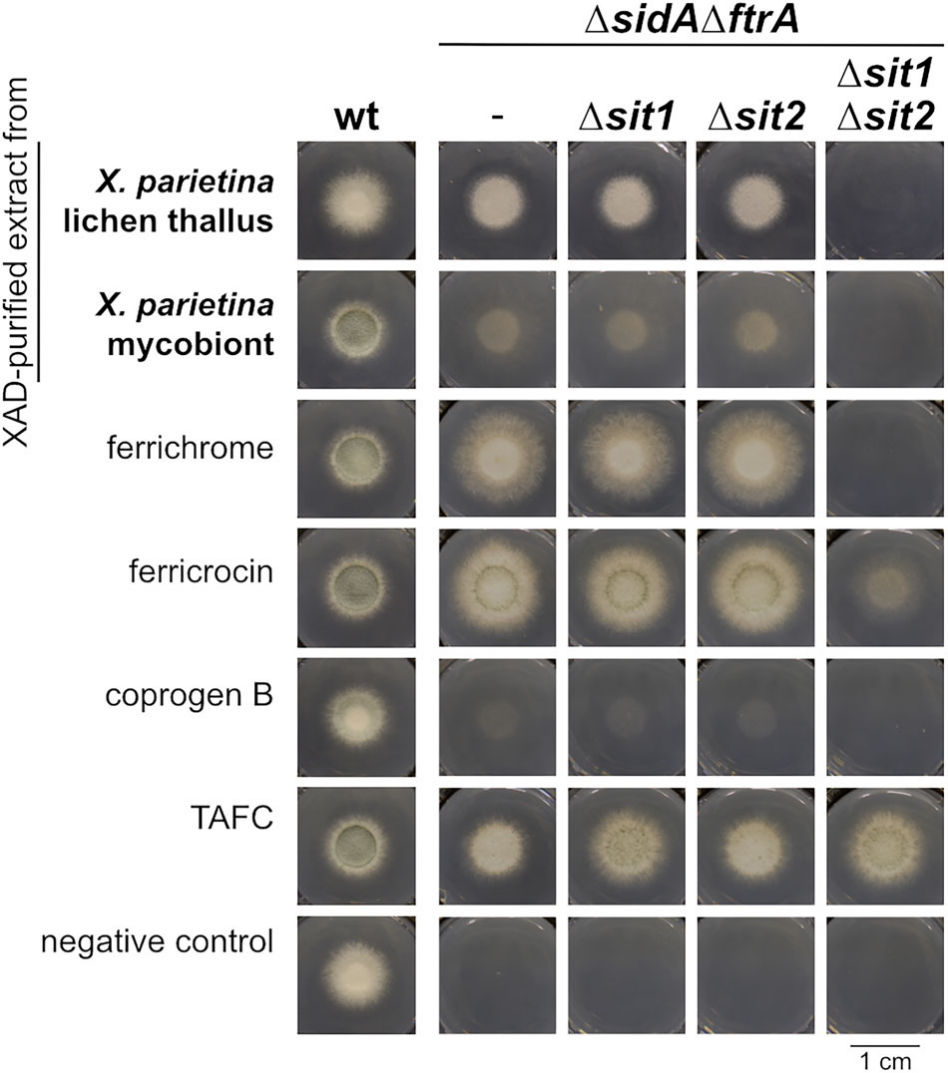
*X. parietina* produces a siderophore that can be utilized by *A. fumigatus.* 10^4^ spores of *A. fumigatus* mutant strains Δ*sidA*Δ*ftrA* (lacking siderophore biosynthesis and RIA), Δ*sidA*Δ*ftrA*Δ*sit1* (lacking additionally Sit1), Δ*sidA*Δ*ftrA*Δ*sit2* (lacking additionally Sit2), Δ*sidA*Δ*ftrA*Δ*sit1*Δ*sit2* (lacking additionally Sit1 and Sit2), and their otherwise isogenetic wild type (wt) were spot-inoculated on solid minimal medium either supplemented with *X. parietina* lichen thallus XAD-purified extract, XAD-purified axenic culture supernatant, ferrichrome, ferricrocin, coprogen B, TAFC or not supplemented (negative control). Control siderophores were used in a final concentration of 1 µM and plates were incubated at 37 °C for 24 hours. Similar results were obtained when the experiment was repeated on three occasions.

We first evaluated siderophore production by *X. parietina* in a bioassay using four previously described *A. fumigatus* mutant strains and their otherwise isogenetic wild type^16^. The Δ*sidA*Δ*ftrA* mutant lacks RIA (Δ*ftrA*) and siderophore biosynthesis (Δ*sidA*) and grows, in contrast to wt, only when supplied with >3 mM ferrous iron or low concentrations of siderophores such as ferricrocin, TAFC, and, to a lesser extent, coprogen. The Δ*sidA*Δ*ftrA*Δ*sit1A*Δ*sit2* mutant is additionally defective in the Sit1 and Sit2 transporters, which are both responsible for uptake of coprogen-type siderophores and show partly overlapping as well as exclusive specificity for different ferrichrome-type siderophores. Thus, this mutant can utilize neither coprogen- nor ferrichrome-type siderophores with exception of a very limited growth response to ferricrocin. Sit1 is the exclusive transporter for ferrioxamine-type siderophores; accordingly, ferrioxamine use can be inferred by comparison with Δ*sidA*Δ*ftrA*Δ*sit1*, Δ*sidA*Δ*ftrA*Δ*sit2* mutant strains. Extracts from both *X. parietina* lichen thallus and the supernatant of *X. parietina* mycobiont liquid culture promoted the growth of all *A. fumigatus* strains, except for Δ*sidA*Δ*ftrA*Δ*sit1*Δ*sit2* (Fig. 1), indicating that *X. parietina* produces either a coprogen- or a ferrichrome-type siderophore in both conditions. The compound is unlikely to be ferricrocin, as limited growth promotion of *sidA*Δ*ftrA*Δ*sit1A*Δ*sit2* would be expected, mirroring the ferricrocin control (Fig. 1). Neither the thallus extract nor the axenic culture supernatant contained ferrioxamine- or fusarinine-type siderophores. The former would promote the growth of the Δ*sidA*Δ*ftrA*Δ*sit2* mutant strain, while the latter would support the growth of all mutant strains used.

### Identification of ferrichrome in *X. parietina* lichen thallus and axenic culture

**Fig. 2:**
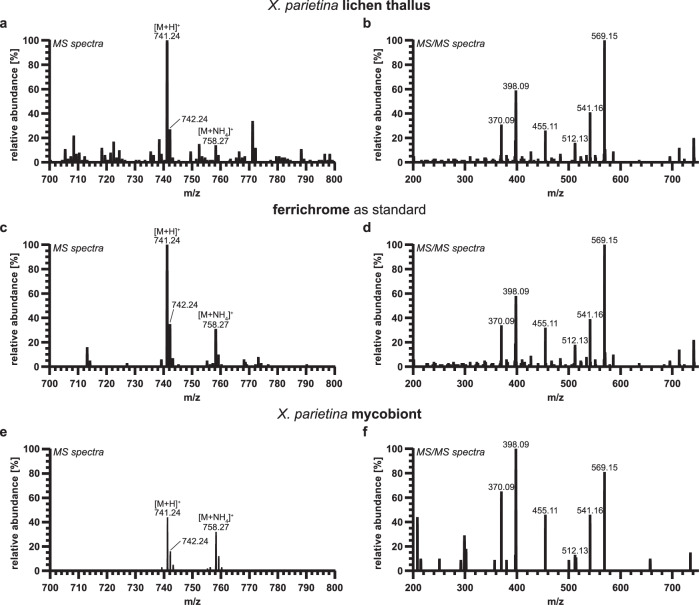
*X. parietina* produces the siderophore ferrichrome as shown by CESI-MS/MS analysis of thallus extracts and axenic culture supernatant. **a** Full MS scan revealing two different adducts M + H^+^ (*m*/*z* = 741.24) and M + NH_4_^+^ (*m*/*z* = 758.27) of ferrichrome and (**b**) MS/MS spectra of ferrichrome (M + H^+^) from *X. parietina* lichen thallus extract. **c** Full MS scan revealing two different adducts M + H^+^ (*m*/*z* = 741.24) and M + NH_4_^+^ (*m*/*z* = 758.27) and (**d**) MS/MS spectra of ferrichrome (M + H^+^) of the ferrichrome reference. **e** Full MS scan revealing two different adducts M + H^+^ (*m*/*z* = 741.24) and M + NH_4_^+^ (*m*/*z* = 758.27) of ferrichrome and (**f**) MS/MS spectra of ferrichrome (M + H^+^) from the supernatant of the axenic culture. Identified ferrichrome MS/MS fragments are 370.09 ([M-AHO-3G-CO]^+^), 398.09 ([M-AHO-3G]^+^), 455.11 ([M-AHO-2G]^+^), 512.13 ([M-AHO-G]^+^), 541.16 ([M-AHO-CO]^+^) and 569.15 ([M-AHO]^+^) with AHO = *N*^*5*^-acetyl-*N*^*5*^-hydroxy- l-ornithine, M = isotopic molecular mass of ferric-ferrichrome and G = glycine. Source data are provided as a Source Data file.

MS analysis of the *X. parietina* lichen thallus extracts identified the ferrichrome-iron chelate (C_27_H_42_FeN_9_O) with two different adducts H^+^ (*m*/*z* = 741.24) and NH_4_^+^ (*m*/*z* = 758.27) (Fig. 2a). The MS/MS fragmentation pattern of this compound (Fig. 2b) matched that of the ferrichrome reference (Fig. 2c, d), in agreement with Essén et al.^40^. While lichen thalli contain a plethora of potentially ferrichrome-producing microorganisms, apart from the major mycobiont^2,41^, MS and MS/MS analyses of the axenic culture supernatant confirmed that *X. parietina* produces ferrichrome (Fig. 2e, f). As indicated by the bioassay (Fig. 1), MS analysis revealed an about 12-fold higher ferrichrome content in the lichen thallus compared to the axenic culture supernatant (1.3 versus 0.11 pmol mg^-1^ dry mass).

### The genetic repertoire of *X. parietina* for SIA, RIA, iron detoxification, and iron regulation

**Fig. 3:**
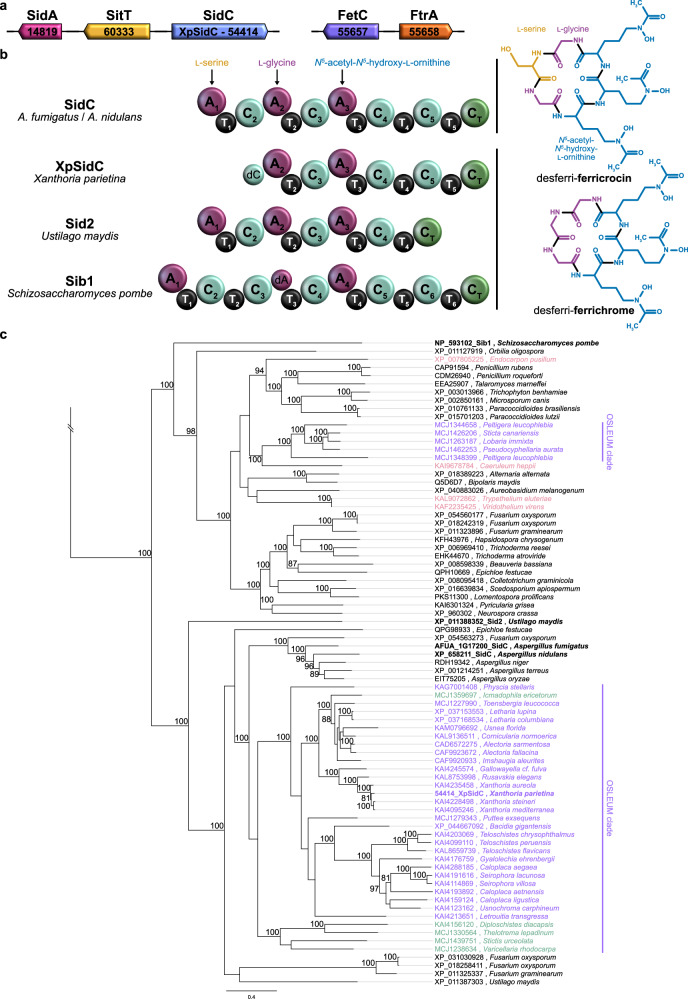
*X. parietina*’s NRPS XpSidC is located in a siderophore biosynthesis gene cluster and shows a reduced domain architecture. **a** Organization of the putative SIA and RIA gene clusters from *X. parietina*. **b** Comparison of the domain architecture of *X. parietina* XpSidC (protein ID 54414) with that of characterized ferrichrome and ferricrocin NRPSs, as well as respective siderophores. Abbreviations: d stands for degenerated, A for adenylation, T for thiolation, C for condensation domains and C_T_ for terminal C domain. The NRPS architecture scheme was inspired by Jenner et al. ^26^. **c** Maximum likelihood phylogenetic analysis of SidC homologs from 41 lichen mycobiont species (Supplementary Data 1) and 30 non-lichenized fungal species known to produce ferrichrome-type siderophores. For clarity, only the clade containing known SidC protein of the best-scoring RAxML tree is shown; the full unrooted tree is provided in Supplementary Fig. S2; bootstrap support values (%) ≥ 80 are indicated at nodes. Proteins discussed in (**b**) are highlighted in bold. Sequences from *Lecanoromycetidae* are shown in violet, *Ostropomycetidae* in turquoise, non-OSLEUM clade (*Chaetothyriomycetidae*, *Dothideomycetes*, and *Leotiomyceta*) in light red, and non-lichenized species in black. The two sequences from *Peltigera leucophlebia* are most likely parts of the same protein (Supplementary Data 1).

Next, we investigated iron homeostasis maintenance in the *X. parietina* mycobiont by systematically analyzing its genome-derived proteome for homologs of *A. fumigatus* proteins known to be involved in this process (Table 1). BLASTp searches revealed homologs to several proteins involved in SIA: The l-ornithine monooxygenase SidA, the ferricrocin NRPS SidC, the ABC-transporter SitT, the acetyltransferase SidL mediating formation of *N*^*5*^-acetyl-*N*^*5*^-hydroxy-l-ornithine (a precursor in ferrichrome-type siderophores), the ferrichrome-type siderophore uptake transporter Sit2 and the functionally uncharacterized SIT family member MirC^19^. Genes encoding SidA, SidT and SidC are clustered in the *X. parietina* genome (Table 1 and Fig. 3a), supporting the involvement of their gene products in a common pathway. In contrast, the *X. parietina* mycobiont lacks homologs of SidI, SidH, the NRPS SidD, which are involved in biosynthesis of fusarinine- and coprogen-type siderophores. In addition, *X. parietina* lacks homologs of the acetyltransferase SidG, which catalyzes formation of TAFC from fusarinine C, and the rhizoferrin biosynthetic enzyme Rfs^23,42^. Taken together, genomic analysis revealed that *X. parietina* contains homologs of all the enzymes required for ferrichrome synthesis while lacking genes for biosynthesis of coprogens, fusarinine-type siderophores and rhizoferrin, consistent with both the bioassay and MS results (Figs. 1 and 2). Therefore, following the *A. fumigatus* siderophore biosynthetic pathway, ferrichrome biosynthesis in *X. parietina* starts with SidA-mediated hydroxylation of l-ornithine, followed by SidL-catalyzed acetylation to form *N*^*5*^-acetyl-*N*^*5*^-hydroxy-l-ornithine and SidC-mediated ferrichrome assembly from three *N*^*5*^-acetyl-*N*^*5*^-hydroxy-l-ornithine moieties and three glycine residues. After secretion and iron chelation, ferrichrome-iron complexes are then most likely taken up by Sit2.

**Table 1:** Putative *X*. *parietina* proteins involved in iron homeostasis identified by BLASTp searches using characterized *A. fumigatus* proteins

*A. fumigatus*				*X. parietina* (mycobiont)					
	name	ID	function	protein ID	E-value	%Ident	%Cov	location	strand
SIA	SidA	Afu2g07680	ornithine monooxygenase	14819	1.97E-50	56.7	74.6	**scaffold_24:151424-152934**	-
	SidC	Afu1g17200	NRPS	54414	1.50E-133	43.8	92.7	**scaffold_24:159416-170008**	+
	SitT	Afu3g03430	ABC transporter	60333	3.95E-127	52.9	131.2	**scaffold_24:153759-157901**	-
	SidL	Afu1g04450	acetyl transferase	57462	1.61E-45	59.8	88.5	scaffold_7:310587-312321	-
	Sit2	Afu7g04730	SIT (ferrichrome)	60974	2.31E-138	42.3	90.9	scaffold_1:1726010-1727758	+
	MirC	Afu2g05730	SIT (?)	70210	0.00E + 00	58.1	91.4	scaffold_19:765893-767976	+
RIA	FtrA	**Afu5g03800**	iron permease	55658	4.36E-93	62.7	86.9	**scaffold_2:1311117-1312490**	+
	FetC	**Afu5g03790**	ferroxidase	55657	0.00E + 00	65	91.6	**scaffold_2:1307800-1309889**	-
	FreB	Afu1g17270	metalloreductase	62215	4.10E-31	45.8	21.8	scaffold_2:1412224-1417032	+
IR	CccA	Afu4g12530	vacuolar iron importer	53727	1.24E-50	48.4	68.8	scaffold_20:290608-291637	+
TF	HapX	Afu5g03920	iron regulator	11971	2.41E-33	53.7	51.3	scaffold_11:588592-590022	-
	SreA	Afu5g11260	iron regulator	11759	5.25E-29	62.8	39	scaffold_10:1126077-1128370	+

The names of species are written in italics. Gene clusters are highlighted in bold. *TF* iron-sensing transcription factors, *IR* iron resistance.

**Fig. 4:**
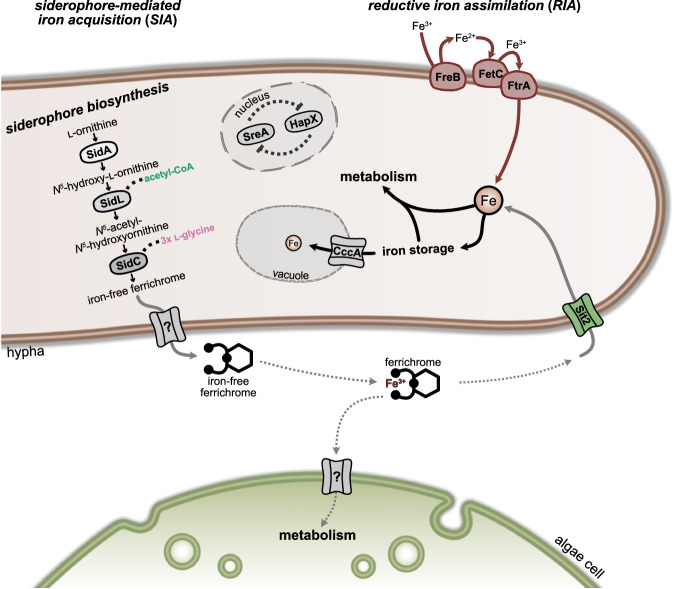
Schematic representation of the iron homeostatic system of the *X. parietina* mycobiont. Explanations are given in the text. The scheme is conceptual and does not aim to capture the full complexity of possible exchange pathways within the symbiosis, including the utilization of RIA by the photobiont.

*X. parietina* also possesses homologs of the metalloreductase FreB, the ferroxidase FetC and the iron permease FtrA (Table 1), indicating the potential for RIA. The genes encoding FetC and FtrA are clustered in the *X. parietina* genome (Table 1 and Fig. 3a), similar to *A. fumigatus*^19^. In addition, *X. parietina* possesses a homolog of the vacuolar iron importer CccA (Table 1), which mediates iron detoxification in *A. fumigatus*^43^. Based on the presence of homologs of SreA and HapX (Table 1), X. *parietina* appears to use a similar iron-sensing and regulating machinery as *A. fumigatus*^7,21,44,45^. A scheme of the proposed iron homeostatic system of the *X. parietina* mycobiont is shown in Fig. 4.

### The *X. parietina* ferrichrome NRPS, XpSidC, is functional in *A. fumigatus* and displays a novel compact domain architecture for ferrichrome synthetases

In agreement with iterative assembly of three distinct precursors, the ferricrocin-synthesizing NRPS SidC from *Aspergillus nidulans* and its *A. fumigatus* ortholog comprise three A-domains (Fig. 3b)^27,28^. As ferrichrome assembly requires only two precursor types, the corresponding NRPS is expected to contain only two A-domains. However, as illustrated in Fig. 3b, ferrichrome NRPSs vary: Sid2 from *Ustilago maydis* contains three A-domains^30^, whereas Sib1 from *Schizosaccharomyces pombe* has two complete and one degenerate A-domain, along with multiple C-domains^29^. The putative *X. parietina* ferrichrome NRPS 54414, here designated XpSidC, exhibits the most compact domain architecture, with only two A-domains, three complete and one degenerate C domain, and a terminal C_T_ domain (Fig. 3b).

**Fig. 5:**
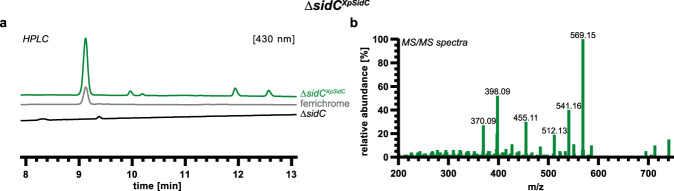
Heterologous expression of *X. parietina*’s NRPS XpSidC in *A. fumigatus* results in the production of ferrichrome. **a** UV-Vis chromatograms of RP-HPLC analysis recorded at 430 nm of Δ*sidC* (black) and Δ*sidC*^*XpSidC*^ (green) mutant extracts and the ferrichrome reference (gray). The main peak of ferrichrome has a retention time of ~9.1 min. **b** MS/MS spectra of ferrichrome (M + H^+^) (*m*/*z* = 741.24) from the Δ*sidC*^*XpSidC*^ mutant extract. Identified ferrichrome MS/MS fragments are 370.09 ([M-AHO-3G-CO]^+^), 398.09 ([M-AHO-3G]^+^), 455.11 ([M-AHO-2G]^+^), 512.13 ([M-AHO-G]^+^), 541.16 ([M-AHO-CO]^+^) and 569.15 ([M-AHO]^+^) with AHO = *N*^*5*^-acetyl-*N*^*5*^-hydroxy- l-ornithine, M = isotopic molecular mass of ferric-ferrichrome and G = glycine. Source data are provided as a Source Data file.

To confirm that XpSidC is indeed able to produce ferrichrome, the encoding gene was expressed under control of the xylose-inducible *xylP* promoter in an *A. fumigatus* Δ*sidC* mutant lacking the NRPS SidC and, consequently, ferrichrome-type siderophores^28^. The resulting mutant strain was termed Δ*sidC*^*XpSidC*^. High-performance liquid chromatography (HPLC) (Fig. 5a) and MS/MS analyses (Fig. 5b) demonstrated production of ferrichrome in mutant strain Δ*sidC*^*XpSidC*^ in contrast to the mutant strain Δ*sidC*, which confirms that XpSidC indeed mediates biosynthesis of ferrichrome.

### *X. parietina* mycobiont genes involved in iron acquisition are up-regulated in lichen thalli grown on concrete compared to tree bark

**Fig. 6:**
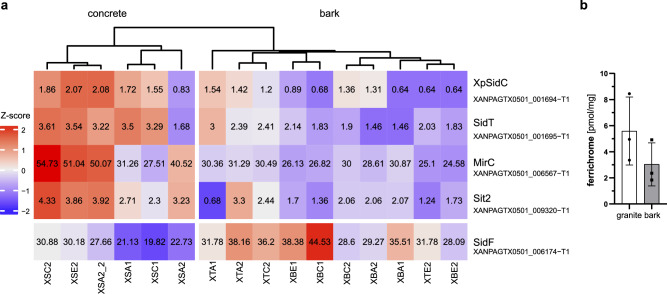
Siderophore production is site-specifically regulated. **a** The gene encoding XpSidC and several other genes potentially involved in iron acquisition are up-regulated in *X. parietina* mycobiont growing on concrete compared to tree bark. The data shown result from a re-analysis of a previously published transcriptomic dataset shown in Supplementary Table S1, for which detailed sample descriptions are provided^41^. Transcriptomic data were pseudoaligned to the predicted transcriptome of *X. parietina* mycobiont with kallisto; differentially expressed genes were identified using sleuth. The heatmap shows expression levels normalized to the mean transcript per million values (TPM) across all samples. Samples were clustered within the substrate groups using hclust function with Euclidean distance and complete linkage method. **b** Ferrichrome content varies widely among *X. parietina* lichen samples from granite and bark. The values shown represent the mean values of biological triplicates, with error bars indicating the standard deviation. The values are provided in Supplementary Table S2.

We identified all iron homeostatic genes listed in Table 1 except FreB, which might have been missed during *de-novo* annotation (Supplementary Table S1) in a recent metatranscriptomic analysis of the *X. parietina* mycobiont of lichen thalli growing on different substrates^41^. Re-analysis of the transcriptomic dataset revealed that the transcript levels of genes encoding XpSidC, along with others that are potentially involved in iron acquisition (e.g., homologs of SitT, MirC and Sit2), are significantly higher expressed on concrete surfaces than on organic tree bark surfaces (Fig. 6a). Interestingly, the gene encoding the SidL homolog was down-regulated in samples from concrete surfaces, in contrast to other siderophore metabolism genes. Notably, the *cccA* gene - expected to exhibit an expression pattern inverse to that of iron acquisition genes - as well as the genes encoding the iron regulators SreA and HapX, did not show differential expression between samples from concrete and organic surfaces (Supplementary Table S1). The single sample collected from metal was excluded from the statistical analysis; its overall expression pattern was similar to the samples from concrete (Supplementary Table S1).

To further investigate potential substrate-dependent ferrichrome production by the *X. parietina* mycobiont, we analyzed ferrichrome contents in three samples growing on *Acer pseudoplatanus* bark and three on granite (Fig. 6b). Ferrichrome content varied 4.7-fold, ranging from 1.8 pmol (1.3 ng) mg^-1^ dry mass in a bark sample to 8.5 pmol (6.3 ng) mg^-1^ dry mass in a granite sample. Although the mean ferrichrome content in granite samples was 1.8-fold higher than in bark samples, the difference was not statistically significant. Nevertheless, these results indicate that ferrichrome levels of *X. parietina* lichen thalli vary substantially across substrates or sites, most likely reflecting differences in iron bioavailability.

### The iron homeostatic genes identified in *X. parietina* are genetically conserved across a wide range of lichen mycobionts

To assess the conservation of iron homeostasis-related proteins identified in *X. parietina* across lichen-forming mycobionts, we analyzed another 53 species with available genome sequences and annotated proteomes, representing 43 genera within the *Pezizomycotina* (Supplementary Data 1). The dataset was dominated by members of the *Lecanoromycetes*/OSLEUM clade (49 species), with additional representation from two *Dothideomycetes* species (*Trypethelium eluteriae, Viridothelium virens*), one *Eurotiomycetes* species (*Endocarpon pusillum*), and one *Leotiomycetes* species (*Caeruleum heppii*). The 49 OSLEUM clade species spanned three subclasses - *Lecanoromycetidae* (34 species), *Ostropomycetidae* (13 species), and *Umbilicariomycetidae* (2 species) - and comprised 12 orders and 19 families. Among these taxa, 26 form macrolichens like *X. parietina*, whereas 25 are crustose lichens.

Among the 53 mycobiont species analyzed, 41 contained a SidC homolog, 41 a SidA homolog, and 51 a SidL homolog (Supplementary Data 1), with all species possessing at least one of these genes. Given potential incompleteness in gene and protein annotations, it is likely that all species are capable of siderophore biosynthesis. No homologs of SidD were detected in any species. The presence of SidC and/or SidL homologs indicates that most, if not all, analyzed mycobionts exclusively produce ferrichrome-type siderophores. In 38 of the 39 species harboring both SidA and SidC homologs, the encoding genes are genomically clustered, as observed in *X. parietina*. 50 species possessed at least one putative siderophore transporter. All 53 species possessed a FetC homolog, and 50 also contained an FtrA homolog, with the corresponding genes genomically clustered, mirroring the organization observed in *X. parietina*. Homologs of FreB and HapX were detected in all species, CccA homologs in 49 species, and SreA homologs in 47 species. Taken together, these findings indicate that the iron homeostatic system is highly conserved across the lichen mycobionts analyzed. There was no obvious difference between macrolichens and crustose lichens

Phylogenetic analysis of the identified 41 mycobiont SidC homologs together with 37 homologs from non-lichenized fungal species known to produce ferrichrome-type siderophores showed that 33 of the 37 OSLEUM-clade sequences form a distinct subclade (Fig. 3c). The remaining four OSLEUM-clade homologs, all from *Peltigerales* species, as well as the four homologs from non-OSLEUM mycobionts (two *Dothideomycetes*, one *Eurotiomycetes* and one *Leotiomyceta*), grouped with sequences from non-lichenized fungi (Fig. 3c). Consistent with this phylogenetic pattern, the 33 OSLEUM-clade homologs in the subclade share the compact NRPS domain architecture characteristic of *X. parietina*, whereas the remaining mycobiont homologs exhibit an architecture more similar to that of *A. fumigatus* (Supplementary Fig. S4). These results support a monophyletic origin of compact-architecture SidC homologs within the OSLEUM clade, with the *Peltigerales* homologs constituting a notable exception.

### Ferrichrome promotes the growth of *T. decolorans*

The potential utilization of siderophore-complexed iron by *T. decolorans* has not been previously described. This microalgae may utilize siderophore-complexed iron through RIA with extracellular reductive release of iron from siderophores similar to fungal RIA^34^. Alternatively, siderophore-complexed iron could be used by siderophore uptake without extracellular iron reduction, as shown for the marine microalgae *Phaeodactylum tricornutum*^46^.

**Fig. 7:**
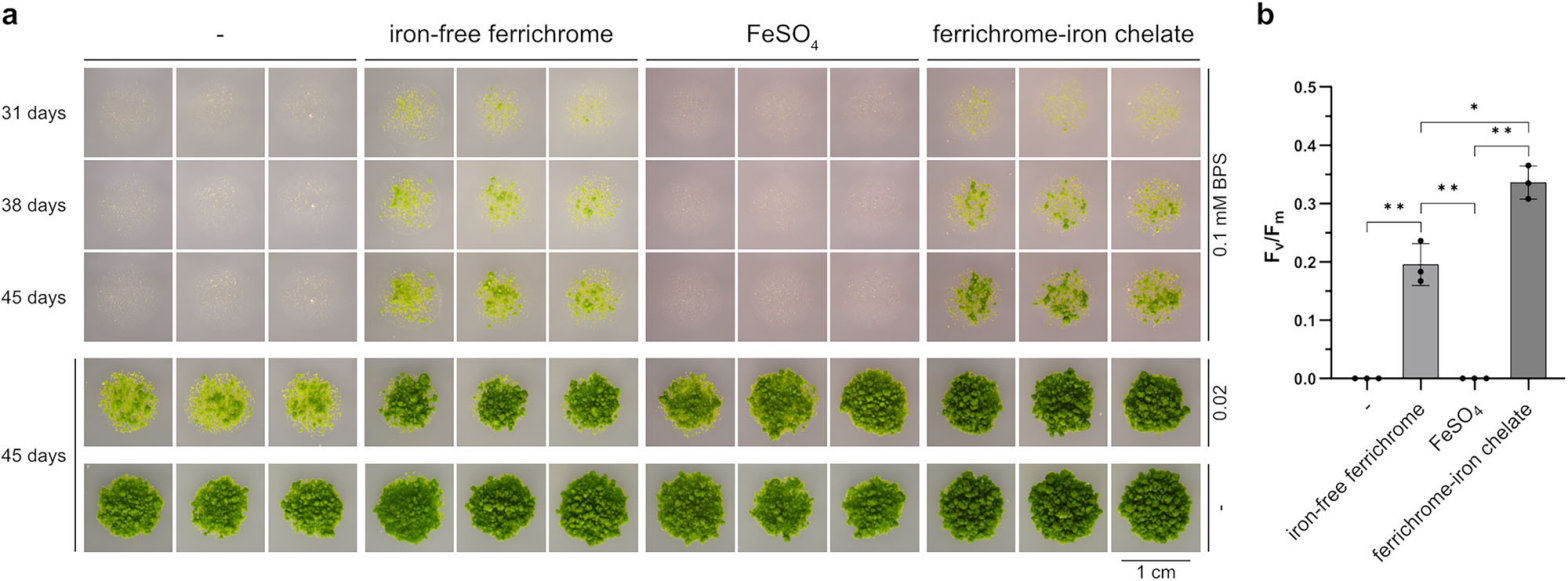
Ferrichrome promotes growth and photosynthesis of *T. decolorans*, the photobiont of *X. parietina.* **a** 10^5 ^*T. decolorans* cells were point-inoculated on solid minimal medium containing 0, 0.02 or 0.1 mM bathophenanthroline disulfonate (BPS), a cell-impermeable, ferrous iron-specific chelator to block iron acquisition involving extracellular reduction of ferric iron. These media were supplemented with either iron-free ferrichrome, FeSO_4_ or ferrichrome-iron chelate to a final concentration of 1 µM each. Pictures of the three-biological replicates (*n* = 3) were taken after incubation at 22 °C with a 12/12 h light/dark regime for the indicated time intervals. **b** Chlorophyll fluorescence analysis by pulse amplitude modulation (PAM), which provides a proxy for photosystem II efficiency (*F*_*v*_*/F*_*m*_), was performed on samples grown in the presence of 0.1 mM BPS after 45 days of incubation and dark adaptation. The values shown represent the mean values of biological triplicates, with error bars indicating the standard deviation. The levels of significance, which were calculated using a one-way ANOVA, are indicated by asterisks (** *p* < 0.0001 and * *p* = 0.0003). No statistical significance was calculated for the -Fe vs. FeSO_4_ comparison. The values are provided in Supplementary Table S4.

To investigate the potential utilization of ferrichrome by *T. decolorans*, 1×10^5^ cells were point-inoculated on solid minimal medium containing 0.1 mM of the cell-impermeable ferrous iron-specific chelator bathophenanthroline disulfonate (BPS) to enhance iron limitation and to block iron acquisition by extracellular reduction^47^. These iron-limited growth media were supplemented with FeSO_4_, iron-free ferrichrome, or ferrichrome-iron chelate to a final concentration of 1 µM each. After 45 days of incubation, *T. decolorans* was largely unable to grow with or without FeSO_4_ supplementation (Fig. 7a). In contrast, the growth of *T. decolorans* was promoted by supplementation with iron-free ferrichrome and, even better, with ferrichrome-iron chelate, (Fig. 7a). The growth promotion of *T. decolorans* by iron-free ferrichrome suggests that even trace amounts of iron chelated in ferrichrome makes iron available to the alga, despite the presence of BPS in the medium. Chlorophyll fluorescence analysis by pulse amplitude modulation (PAM), i.e., the determination of the maximum quantum yield of photosystem II (*F*_*v*_*/F*_*m*_) as an indicator for the photosynthetic efficiency, confirmed the beneficial effect of ferrichrome supplementation, again with higher values for cultures treated with the ferrichrome-iron complex compared to iron-free ferrichrome (Fig. 7b). These data indicate that *T. decolorans* is able to utilize ferrichrome, most likely without extracellular reductive release of iron from the siderophore.

Consistent with growth reduction by BPS-mediated iron limitation, algal growth increased upon a five-fold reduction in BPS concentration and was further enhanced when BPS was omitted entirely. Under these conditions, the growth-promoting effects of both iron-free ferrichrome and the ferrichrome-iron complex are only marginal, likely due to uptake of iron via RIA in the absence of BPS.

### Bioinformatic analysis reveals potential iron acquisition systems of *Trebouxia* sp

Diverse algae can acquire siderophore-bound iron through either reductive or non-reductive pathways. The freshwater microalga *Chlamydomonas reinhardtii* (*Chlorophyta*, like *Trebouxia* spp.) employs RIA involving metalloreductases, a ferroxidase and an iron permease that are homologous to *A. fumigatus* FreB. FetC and FtrA, respectively^34,48,49^. In contrast, the diatom *P. tricornutum* (*Stramenopiles*) internalizes ferrioxamine B- and ferrichrome-bound iron through ISIP1-dependent endocytosis following binding to the FBP1 protein, with intracellular iron release mediated by reductases homologous to fungal FreB^46^. The marine green alga *Dunaliella salina* (*Chlorophyta*) appears to combine both strategies, encoding FRE1 (fungal FreB-like) and FOX1 (fungal FetC-like) for putative RIA but lacking FtrA-like permeases^50^. Moreover, it possesses transferrin-like (TTF-1) and ISIP1-like (SUPT) proteins indicating endocytotic siderophore uptake, which are co-localized with FOX1 at the cell surface and up-regulated under iron limitation transferrin homologs^50,51^. BLASTp analyses indicate that different *Trebouxia* sp. isolates harbor homologs of FreB, FetC, and FtrA, suggesting fungal-like RIA, and in some species, ISIP1/SUPT and TTF-1 homologs, implying potential for non-reductive siderophore uptake (Table 2 and Supplementary Table S3).

**Table 2:** Proteins potentially involved in iron acquisition in *Trebouxia* sp

Query: *A. fumigatus* metalloreductase FreB; Q4WR75.2; 743 amino acids					
*Trebouxia* isolate	Query Cover (%)	E value	Per. Ident.	Acc. Len.	Accession
*Trebouxia* sp. C0006	62 %	6.00E-108	22.82	748	KAL0043219.1
*Trebouxia* sp. C0009 RCD-2024	59 %	5.00E-104	19.31	799	KAL3146993.1
*Trebouxia* sp. C0010 RCD-2024	59 %	2.00E-102	19.52	815	KAL3134756.1
*Trebouxia* sp. C0004	57 %	9.00E-78	16.82	755	KAL0041029.1
*Trebouxia* sp. C0005	57 %	1.00E-75	16.82	721	KAL0043585.1
*Trebouxia* sp. A1-2	57 %	2.00E-74	16.82	813	KAA6424729.1
Query: ***A. fumigatus*** **ferroxidase FetC;** E9R598.1; 592 amino acids					
*Trebouxia* sp. C0004	83 %	4.00E-152	24.32	751	KAL0024990.1
*Trebouxia* sp. C0009 RCD-2024	53 %	2.00E-106	25.95	571	KAL3136665.1
*Trebouxia* sp. C0005	70 %	1.00E-105	26.84	691	KAL0038686.1
*Trebouxia* sp. C0006	67 %	7.00E-104	27.74	752	KAL0043919.1
*Trebouxia* sp. C0010 RCD-2024	43 %	3.00E-101	25	374	KAL3145498.1
*Trebouxia* sp. A1-2	23 %	3.00E-37	32.48	374	KAA6419185.1
*Trebouxia lynnae*	56 %	2.00E-04	17.63	718	QOL01226.1
Query: ***A. fumigatus*** **high-affinity iron transporter FtrA;** XP_747964.2; 370 amino acids					
*Trebouxia* sp. C0006	96 %	2.00E-148	30.85	584	KAL0047096.1
*Trebouxia* sp. C0004	98 %	2.00E-140	31.06	390	KAL0029032.1
*Trebouxia* sp. C0010 RCD-2024	92 %	5.00E-135	32.26	392	KAL3141519.1
*Trebouxia* sp. C0005	89 %	9.00E-128	31.42	324	KAL0018724.1
*Trebouxia* sp. C0009 RCD-2024	92 %	2.00E-127	31.87	392	KAL3155144.1
*Trebouxia* sp. A1-2	86 %	1.00E-96	30.55	313	KAA6417637.1
Query: ***P. tricornutum*** **ISIP1;** XP_002183904.1; 569 amino acids					
*Trebouxia lynnae*	53 %	3.00E-77	16.72	634	QOL01286.1
*Trebouxia* sp. C0010 RCD-2024	38 %	3.00E-72	16.74	540	KAL3150830.1
*Trebouxia* sp. C0004	39 %	7.00E-68	18.91	529	KAL0017780.1
*Trebouxia* sp. C0009 RCD-2024	34 %	2.00E-53	17.73	485	KAL3137606.1
Query: ***D. salina*** **transferrin-like protein TTF-1;** AAB36531.1; 1274 amino acids					
*Trebouxia* sp. C0010 RCD-2024	91 %	0	22.59	769	KAL3141978.1
*Trebouxia* sp. C0009 RCD-2024	91 %	0	22.8	767	KAL3153465.1

The names of species are written in italics.

PSI-BLAST (Position-Specific Iterated BLAST) searches were conducted with 2 iterations in the NCBI database^76^. Query proteins are given in the headers. If a *Trebouxia* sp. isolate contained multiple homologs, only the homolog with the highest significance (lowest E-value) is shown. The complete analysis is provided in Supplementary Table S3.

## Discussion

Lichens can grow on bare rock, bark or soil, and have long been recognized as agents of mineral weathering and soil stabilization^52,53^. The species name of *X. parietina* itself is derived from the Latin word *paries*, for wall, because the lichen often grows on walls and rocks. For most fungal species, siderophore-mediated iron acquisition plays an important role in growth and interaction with other organisms^7^, but its potential role in lichens remained largely unknown^54^, despite its potential significance to the lichen symbiosis. Siderophore-specific NEXAFS signatures provided by X-ray spectromicroscopy, localized in close proximity to *Parmelia saxatilis* hyphae, suggested siderophore production by the lichen thallus, but its origin remained unclear^55^. The present study provides evidence for synthesis of a hydroxamate-type siderophore, ferrichrome, by a lichen mycobiont both in lichen thallus and the supernatant of an axenic culture (Figs. 1 and 2). The ferrichrome content of the lichen thallus was ~12-fold higher than in the axenic mycobiont culture. This difference may arise from multiple factors such as the presence of additional ferrichrome-producing organisms in the lichen thallus or an upregulation of ferrichrome biosynthesis in the symbiotic state. The axenic culture represents a highly artificial condition and the transition to the symbiotic state has previously been shown to be accompanied by shifts in metabolite accumulation between free-living fungi in culture and their lichenized counterparts, as exemplified for photosynthetic pigments and antioxidants^56^. Nevertheless, inclusion of the axenic culture unequivocally demonstrates that the mycobiont itself is capable of producing ferrichrome in the absence of other organisms – something that cannot yet be resolved in the natural thallus. Future studies would benefit from direct transcriptomic comparisons of matched axenic and symbiotic datasets generated under strictly controlled and comparable conditions.

Ferrichrome is the simplest ferrichrome-type siderophore built from only two different moieties, three glycine and three *N*^*5*^-acetyl-*N*^*5*^-hydroxy-l-ornithine residues (Fig. 3). Mining of the *X. parietina* genome identified putative genes involved in SIA, RIA, iron detoxification and iron regulation (Table 1 and Fig. 4). Functional characterization of lichen genes is challenging due to the lack of established genetic engineering methods including gene deletion. An alternative is heterologous gene expression in suitable host organisms, but even this has proved challenging^57^ and there are only few successful examples^58,59^. Here, the *X. parietina* ferrichrome NRPS XpSidC has been confirmed to synthesize ferrichrome through heterologous expression in *A. fumigatus* (Fig. 5). BLASTp searches and an amino acid alignment demonstrated high similarity between *X. parietina* XpSidC and *A. fumigatus* SidC except for the absence of N-terminal A (A_1_)- and T (T_1_)-domains and the majority of N-terminal C (C_1_) domain in *X. parietina* XpSidC compared to *A. fumigatus* SidC (Supplementary Fig. S3). Notably, this N-terminal module was shown to mediate the incorporation of serine for assembly of ferricrocin^26^, which is not required for ferrichrome biosynthesis. The following A-domains (A_1_ and A_2_) of *A. fumigatus/A. nidulans* SidC, which are highly conserved in *X. parietina* XpSidC (Supplementary Fig. S3), were shown to mediate loading with glycine and *N*^*5*^-acetyl-*N*^*5*^-hydroxy-l-ornithine^26^. These results may indicate that *X. parietina* XpSidC evolved from an NRPS with a SidC architecture by loss of the N-terminal module. In summary, *X. parietina* XpSidC displays the most condensed architecture known for any ferrichrome NRPS.

Homology searches revealed that genes involved in SIA, RIA, iron detoxification and iron regulation are conserved across a wide range of lichen mycobionts, both within and beyond the OSLEUM clade (Supplementary Fig. S3). Phylogenetic analysis (Fig. 3c) and protein domain architecture (Supplementary Fig. S4) support a monophyletic origin of compact-architecture SidC homologs within the OSLEUM clade, with the *Peltigerales* homologs representing a notable exception.

The gene encoding XpSidC, along with several other genes that are potentially involved in iron acquisition, was found to be up-regulated in lichen thalli growing on concrete surfaces as opposed to organic surfaces, such as tree bark (Fig. 6a), which may reflect the greater challenge lichens face in acquiring iron from inorganic surfaces as opposed to organic surfaces containing biologically processed iron sources. Surprisingly, the gene encoding the siderophore biosynthetic enzyme SidL exhibited regulation opposite to that of SidC, suggesting that it may be subject to regulatory inputs beyond iron. Ferrichrome levels in *X. parietina* thalli varied 4.7-fold across bark- and granite-grown substrates, (Fig. 6b). Although granite samples averaged 1.8-fold higher than bark, the difference was not statistically significant. These results suggest, however, that ferrichrome production varies substantially between substrates, likely reflecting differences in iron bioavailability.

In the *X. parietina* thallus, the photobiont partner *T. decolorans* is embedded in a hyphal mycobiont cortex and is dependent on nutrient supply from the mycobiont. As a photosynthetic organism that plays a vital role in providing the mycobiont with carbon sources, it is essential that the mycobiont provides sufficient iron for processes such as photosynthesis. This raised the question of whether *T. decolorans* could utilize ferrichrome produced by the mycobiont.

Algae can acquire siderophore-bound iron through either reductive or non-reductive pathways. In the freshwater microalga *Chlamydomonas reinhardtii* (*Chlorophyta*, like *Trebouxia* spp.), RIA involves metalloreductases that extracellularly release iron from siderophores, followed by uptake via the ferroxidase Fox1, the permease Ftr1, and likely FreB-like reductases, all homologous to fungal proteins^34,48,49^. In contrast, the diatom *P. tricornutum* (*Stramenopiles*) internalizes ferrioxamine B- and ferrichrome-bound iron through ISIP1-dependent endocytosis following binding to FBP1, with intracellular iron release mediated by reductases homologous to fungal FreB^46^. The marine green alga *Dunaliella* spp. (*Chlorophyta*) appears to combine both strategies, encoding FRE1 (FreB-like) and FOX1 (FetC-like) for putative RIA but lacking FtrA-like permeases^50^. Moreover, it possesses transferrin-like (TTF-1) and ISIP1-like (SUPT) proteins, indicating endocytotic siderophore uptake; these proteins co-localize with FOX1 at the cell surface and up-regulated under iron limitation transferrin homologs^50,51^. BLASTp analyses indicate that *Trebouxia* spp. isolates harbor homologs of FreB, FetC, and FtrA, suggesting fungal-like RIA, and in some species, ISIP1/SUPT and TTF-1 homologs, implying potential for non-reductive siderophore uptake, while FBP1 homologs were not found (Table 2, Supplementary Table S3). Given the pronounced genomic diversity within *Trebouxia*^60^ and the frequent coexistence of multiple photobiont lineages within single lichens^61,62^, such metabolic versatility may enhance symbiotic adaptability. Notably, BLASTp searches revealed that *Trebouxia* species as well as *C. reinhardtii, Dunaliella* species and *P. tricornutum* lack homologs of fungal siderophore transporters.

Our axenic growth studies indicate that *T. decolorans* acquires iron via two distinct routes. First, iron may be obtained through the direct uptake of siderophore–iron complexes, potentially mediated by homologs of *P. tricornutum* ISIP1 or the transferrin-like protein TTF-1 from *Dunaliella salina* (Table 2; Supplementary Table S3). Consistent with this, ferrichrome was sufficient to overcome the axenic growth restriction of *T. decolorans* imposed by BPS, an inhibitor of reductive iron assimilation (RIA) (Fig. 7). Second, *T. decolorans* most likely also acquires iron via RIA, as supported by the presence of homologs of FreB, FetC, and FtrA (Table 2; Supplementary Table S3). In agreement with this, alleviation of iron limitation - either by reducing or omitting BPS - resulted in a significant increase in axenic growth of *T. decolorans* (Fig. 7).

Additional evidence that *T. decolorans* can utilize ferrichrome is provided by the fact that ferrichrome is produced in its iron-free form and exhibits an exceptionally high affinity for ferric iron (logK_f_ = 29.1). Consequently, such chelators inhibit the growth of organisms unable to utilize them by sequestering environmental iron, even from BPS, and thereby inducing iron starvation. Consistently, even iron-free ferrichrome promoted *T. decolorans* growth in the presence of BPS (Fig. 7). Thus, the utilization of mycobiont-produced ferrichrome by the photobiont appears to be a basic requirement for mycobiont-photobiont mutualism in this lichen. Collectively, the *X. parietina* mycobiont produces a siderophore accessible to both partners, supporting mutualistic iron sharing within the lichen symbiosis (Fig. 4). At present, direct physiological analysis of iron handling in the lichen thallus is lacking; future studies incorporating such approaches, including spatial visualization of ferrichrome in intact thalli, would be informative. As lichens comprise, alongside the mycobiont and photobiont, a diversity of potentially siderophore-producing microorganisms, these may contribute to the iron supply of both symbiotic partners.

Taken together, iron acquisition by both the mycobiont and the photobiont mediated by mycobiont-produced ferrichrome represents another example of metabolic exchange between lichen symbionts besides carbon, phosphorus, and nitrogen between symbionts^63^. Moreover, this work extends our appreciation of fungal siderophores as versatile mediators of biotic interactions, capable of fostering mutualistic associations, as demonstrated here, while also driving microbial competition^64^ and the virulence of pathogens^47^.

## Methods

### *Xanthoria parietina* collections from Austria

*X. parietina* samples are listed in Supplementary Table S5.

### Axenic culture of the *X. parietina* mycobiont

The mycobiont strain L 2379 of *X parietina* (L.) Th. Fr. from the culture collection of the University of Trieste, Italy, derived from a single-spore isolate, was used^65^. Species identity was confirmed using ITS sequencing (NCBI GenBank accession number: MT513231). The fungal mycelium was grown in 50 mL of modified liquid Lilly-Barnett Medium (mLBM) at pH 5.0, supplemented with 20 g sucrose L^-1^ and iron availability adjusted to 0.2 mg of Fe(NO_3_)_3_ nonahydrate L^-1^ ^66^. The fungal cultures were incubated in a growth chamber (Percival PGC-6HO, CLF Plant Climatics GmbH, Wertingen, Germany) at 20 °C under a 14/10 h light/dark regime with an illumination intensity of 20 μmol photons m^−2^s^−1^ ^65,67^. The cultures were grown for ≥ 8 months. At regular intervals, the culture medium was replaced by fresh sterile nutrient solution as suggested by Yoshimura et al. ^66^. After three to four months of mycobiont growth, 40 ml of the culture supernatant was harvested regularly and the residual mycobiont biomass was separated by centrifugation at 1000 *g* for two minutes. The culture supernatant was then analyzed for the presence of siderophores.

### Axenic culture of *T. decolorans*, the *X. parietina* photobiont

The algal strain *T. decolorans* (ITS Genbank accession number MT603980) was obtained from the culture collection of the University of Trieste^65^. Notably, the axenic cultures of *X. parietina* and *T. decolorans* were not derived from the same thallus. Axenic algal stock cultures were grown on solid minimal medium (MM) containing 1% (*w*/*v*) glucose and 20 mM glutamine as carbon and nitrogen sources, respectively^68^. Cultures were^69^ incubated at 22 °C with a 12/12 h light/dark regime. According to its exponential growth^69^, algal cultures were harvested after 3 weeks by scraping off the cells with a sterile inoculation loop and placed in a 0.05% Tween80 solution. The culture suspension was vortexed to break up any cell clumps, after which it was pelleted by centrifugation at 939 *x g* for 2 minutes at room temperature. The supernatant was discarded, and the cell pellet was washed by resuspension in a 0.05% Tween80 solution. The cell count of this suspension was determined using a Thoma cell counting chamber. Growth assays were performed in 6-well plates (CC7672-7506 - CytoOne**®**, Starlab GmbH, Germany) containing 6 mL of solid MM with the indicated concentrations of bathophenanthroline disulfonate (BPS) with or without supplementation with iron, iron-free siderophores or siderophore-iron chelates. For this, 10^5 ^*T. decolorans* cells were point-inoculated onto media and the cultures were incubated at 22 °C with a 12/12 h light/dark regime for the indicated time periods. After 45 days of incubation, measurements of chlorophyll fluorescence were conducted with an Imaging PAM CCD camera (M-series, Walz, Germany). For determination of maximum quantum yield of photosystem II (*F*_v_/*F*_m_), as calculated *via* (*F*_m_*-F*_o_)/*F*_m_, a 600 ms saturating pulse was applied to measure maximum fluorescence (*F*_m_). Background fluorescence (*F*_o_) was measured at 8 Hz. All samples were dark-adapted for 1 h before measurement. *F*_*v*_*/F*_*m*_ was measured for each of the 3 biological replicates through the lid of the 6-well plates to maintain sterility.

### *Aspergillus fumigatus* growth conditions

For spore production and bioassays, *A. fumigatus* strains were grown on solid MM^68^ containing 1% (*w*/*v*) glucose and 20 mM glutamine as carbon and nitrogen sources, respectively. For iron-depleted conditions, iron was omitted. For spore production of *A. fumigatus* strains, media were supplemented to a final concentration of 0.03 mM FeSO_4_; for *A. fumigatus* with a Δ*sidA*Δ*ftrA* mutant background^16^, media were supplemented to a final concentration of 5 mM FeSO_4_. For point inoculation on solid plates, 10^4^ spores were used and incubated at 37 °C. Liquid-shaking cultures containing 100 mL of MM with 1% xylose for the induction of the P*xylP* promoter were inoculated with 10^6^ spores per mL. The cultures were then incubated at 200 rpm for 18 h at 37 °, followed by incubation at 20 °C for three days. The media for the bioassays were cooled to ~60 °C before siderophores or the extracts to be tested were added. The *A. fumigatus* strains used in this study are listed in Supplementary Table S5.

### *X. parietina* lichen thallus gDNA isolation for gene amplification by PCR

To isolate gDNA from the lichen thallus, 50 mg of biomass was placed in a 2 mL safe-lock tube with three carbide beads (∅ 3 mm - QIAGEN) and homogenized for 2 min at 30 Hz using a mixer mill (MM 400 - RETSCH GmbH). After addition of 300 μL of a cell lysis solution (5 mL 1 M Tris (pH 7), 30 mL 10% SDS, 10 mL 0.5 M EDTA (pH 8), and 54 mL distilled H_2_O), the tubes were returned to the mixer mill for 2 minutes at 30 Hz. After incubation on a benchtop speed shaker at 2700 rpm for 15 min, 15 μL RNase A solution [10 mg mL^-1^] was added followed by incubation at 65 °C for 1 hour. After incubation on ice for 5 min and addition of 150 μL of MPC Protein Precipitation Reagent (No. 158910 - QIAGEN Sciences, Maryland, US), the tubes were vortexed and centrifuged at 16,090 *x g* for 10 min at room temperature. 300 μL of the supernatant was transferred to a clean 1.5 mL tube and mixed with an equal volume of isopropanol by inversion and incubated for 10 min at room temperature. The precipitated DNA was then pelleted by centrifugation at 16,090 *x g* for 10 min at room temperature. The DNA was briefly washed twice with 1 mL 70 % ethanol, centrifuged and the supernatant discarded. The DNA pellet was air dried for 10 min and then resuspended in 20 μL of H_2_O and stored at -20 °C until further use.

### Generation of *A. fumigatus* strain Δ*sidC*^*XpSidC*^

The P*xylP* promoter was utilized for the conditional expression of XpSidC (*X. parietina* protein ID 54414). To achieve this, the pSO16 plasmid was amplified to be used as a plasmid backbone for marker-free transformation targeting the *fcyB* locus^70–72^. This plasmid backbone includes the P*xylP* promoter, which allows strong induction in the presence of xylose while maintaining minimal basal expression in its absence, as well as the 5′- and 3′- non-coding regions of *fcyB* for targeted genomic integration into the *fcyB* locus^70–72^. The backbone was amplified by PCR using primers SO066 (5´-TGTGAAATTGTTATCCGCTC) and SO067 (5´-GGTTGGTTCTTCGAGTCG), resulting in a product with a fragment length of 6660 bp. A fragment containing the coding sequence and a 400 bp 3’-non-coding region of XpSidC was amplified from gDNA, isolated from the *X. parietina* thallus, by PCR using primers IH137 (5´-atcgactcgaagaaccaaccATGGATTTCCCTTCCTTG) and IH138 (5´-gagcggataacaatttcacaTACTGACATTGAGACTGTC), resulting in a product fragment length of 11,033 bp. The PCR products were column purified and then assembled using the NEBuilder HiFi DNA assembly reaction (New England Biolabs, Inc. Ipswich, MA, USA), resulting in the plasmid, termed pIH032. For fungal transformation, the plasmid was linearized by digestion with the restriction enzyme *Not*I (New England Biolabs, Inc. Ipswich, MA, USA). Integration at the *fcyB* locus was selected by supplementation of 5-fluorocytosine with a final concentration of 10 μg/mL^71^. The Δ*sidC* mutant of *A. fumigatus*, which is unable to produce ferrichrome-type siderophores^28^, was used as the recipient strain for genetic transformation using protoplasts^73^, resulting in a mutant termed Δ*sidC*^*XpSidC*^. Transformation was confirmed by Southern blot analysis Supplementary Fig. S5.

### Siderophore extraction from lichen thallus, the supernatant of axenic *X. parietina* mycobiont culture, and from mycelia of the *A. fumigatus* Δ*sidC*^*XpSidC*^ mutant

After four months of growth, 40 mL of the supernatant from an axenic *X. parietina* mycobiont culture was harvested by separation from the mycobiont biomass by centrifugation at 1000 *g* for 2 min. To saturate siderophores with iron, FeCl_3_ was added to a final concentration of 400 µM. Chromatographic purification of siderophores from the supernatant was performed according to the method previously described by Oberegger et al. ^74^. Briefly, 10 mL of the supernatant from the axenic mycobiont culture was purified using Amberlite™ XAD-16N resin chromatography. The eluate was dried under a constant airflow, resuspended in 1 mL distilled H_2_O. For the bioassay (see below), 20 μL of this XAD extract was utilized for each mutant. For mass spectrometry (MS), 200 µL of the XAD-purified mycobiont supernatant was applied onto a C18 ISOLUTE® column (Part No. 220-0005-A, Biotage, Sweden) and washed twice with distilled H_2_O. Siderophores were eluted with 200 µL methanol, air-dried and suspended in 200 µL distilled H_2_O.

To isolate siderophores from the lichen thallus, 4.8 g of biomass was homogenized with steel beads (∅ 2.5 cm) for 15 s at 30 Hz using a mixer mill (MM 400 - RETSCH GmbH). The homogenized thalli were resuspended in 200 mL of distilled H_2_O, stirred for 30 min, and sonicated four times in an ultrasonic bath for 2 min. The chromatographic purification of siderophores was performed according to Oberegger et al. ^74^, with the following modifications: a chromatographic column with frit and beaded rim (∅ 3 cm) was packed with Amberlite™ XAD-16N resin (15 cm height, swollen in distilled H_2_O; cas. no. 9003-69-4; Sigma-Aldrich Inc., USA) and rinsed with 250 mL distilled H_2_O. FeCl_3_ was added to the thallus suspension to a final concentration of 400 µM to saturate siderophores with iron, followed by centrifugation (2113 *g* for 10 min) to remove cellular debris. The thallus suspension was applied to the XAD-16 resin column. The column was washed with 250 mL distilled H_2_O. The siderophores bound to the column by hydrophobic interaction were eluted with 200 mL methanol. The sample was then dried under constant airflow and resuspended in 2 mL distilled H_2_O. For the bioassay (see below), 20 μL of this XAD extract was utilized for each mutant. For the MS analysis, 200 µL of the XAD-purified lichen thallus extract was applied onto a C18 ISOLUTE® column (Part No. 220-0005-A, Biotage, Sweden) and washed twice with distilled H_2_O. Siderophores were eluted with 200 µL methanol, air-dried and suspended in 200 µL distilled H_2_O.

To isolate the intracellular siderophores from the *A. fumigatus* Δ*sidC*^*XpSidC*^ mutant, 2.5 g of freeze-dried biomass was extracted as described for the *X. parietina* lichen thallus extraction. Subsequently, samples were subjected to reversed-phase high-performance liquid chromatography (RP-HPLC) and MS analyses.

### *A. fumigatus* mutant-based bioassay for siderophore detection

The bioassays were performed in 12-well (CC7672-7512 - CytoOne**®**, Starlab GmbH, Germany) plates containing 1.5 mL solid MM and 20 µL of the XAD-purified samples of the axenic *X. parietina* mycobiont or lichen thallus per well. *A. fumigatus* mutant strains that can only grow in the presence of specific siderophores and allow the differentiation between siderophore types, were point-inoculated onto the media and incubated at 37 °C for 24 hours. As a control, plates supplemented with different siderophores or lacking siderophores were used.

### MS **analysis of siderophores**

For MS analysis a CESI 8000 Plus capillary electrophoresis system (Sciex, Brea, CA, USA) equipped with a bare fused-silica capillary (total length: 90 cm, i.d.: 30 μm, o.d.: 150 μm) was coupled to a Q-Exactive HF mass spectrometer (Thermo Scientific, Bremen, Germany). A terminal porous segment of the bare fused-silica capillary inside the sprayer interface acted as nanospray emitter; a secondary capillary filled with conductive liquid enabled the electric contact. The background electrolyte (BGE) used for CE was 0.1% (*v/v*) formic acid. Prior to each analysis, the conductive liquid capillary was rinsed with BGE for 1 min at 100 psi; the separation capillary was rinsed with methanol for 2 minutes followed by BGE for 3 min at 100 psi. Samples were injected by applying a pressure of 5 psi for up to 15 sec, which corresponds to a sample volume of 12.5 nL. The separation was performed at +25 kV with a pressure of 3 psi applied at the capillary inlet assisting the transport of analytes towards the MS. This method was previously applied to the analysis of TAFC^75^.

The mass spectrometer was operating in data-independent acquisition mode: Full scan spectra were acquired at a resolution of 240,000 (at *m/z* = 200) using a scan range of *m/z* = 300–2000. Automatic gain control target was set to 3e6 with a maximum ionization time of 200 ms. For data-independent MS/MS acquisition ions at *m/z* = 490.13 (rhizoferrin), 741.24 (ferrichrome + H^+^), 758.27 (ferrichrome + NH_4_^+^) were isolated and fragmented using higher-energy collisional dissociation at five different normalized collision energies ranging from 10 to 34. The isolation window was set to *m/z* = 2.0 and fragment spectra were acquired at a resolution of R = 15,000 with the automatic gain control target set to 2e5.

For data analysis, the three *m/z* values mentioned above were extracted from full scans using Qual Browser software (part of Thermo Xcalibur 4.2.47). Mass tolerance for ion extraction was set to 10 ppm. Peak areas were integrated using Qual Browser software. To quantify the amount of ferrichrome in the samples, the ferrichrome standard was diluted to 100 µM and 1 µM, respectively. Both standard dilutions were analyzed, and the resulting peak areas were used to establish a two-point calibration curve. Based on this calibration, the amount of ferrichrome present in the prepared samples was determined. Each sample was analyzed once.

### Chromatographic siderophore analysis

Reversed-phase HPLC analyses of a ferrichrome reference (100 µg mL^-1^ aqueous solution) and of the test samples were performed using an UltiMate 3000 RS UHPLC pump, UltiMate 3000 auto sampler, UltiMate 3000 column compartment (Thermo Fisher Scientific, Vienna, Austria) with column oven temperature set to 25 °C. A Jupiter Proteo C12 4 µM 250 × 4.6 mm column (Part No. 00G-4396-E0; Phenomenex, Aschaffenburg, Germany) was used as stationary phase with 0.1 % TFA in water (A) and 0.1 % TFA in acetonitrile (B) as mobile phases with a flow rate of 1.0 mL min^-1^ and the following gradient: 10 % B (0 - 3 min); 10 % - 60 % B (3 - 16 min); 60 % B (16 - 18 min). UV-Vis detection was carried out with an UltiMate 3000 Diode Array Detector (Thermo Fisher Scientific, Vienna, Austria) at λ = 430 nm to detect orange-colored compounds such as iron-saturated siderophores.

### Siderophores

TAFC and ferricrocin were produced and isolated *in-house* from iron-starved *A. fumigatus* liquid cultures. Briefly, *A. fumigatus* was grown in 1 L of liquid MM (10^6^ spores/mL) for 18 h at 37 °C with shaking at 200 rpm. The culture was filtered to separate the supernatant from the mycelium, which was then lyophilized. To saturate TAFC with iron, the supernatant was adjusted to 1 mM FeCl_3_ and applied to an Amberlite^™^ XAD-16N resin column (3 cm diameter, 15 cm height; Sigma-Aldrich, CAS 9003-69-4) pre-equilibrated with distilled water. The column was washed with 1 L of distilled water and then eluted with 100 mL of methanol. The eluate was evaporated to dryness, reconstituted in 100 mL water, and TAFC was extracted three times with equal volumes of chloroform. To purify ferricrocin, the lyophilized mycelium was pulverized using a mixer mill (MM 400 - RETSCH GmbH) at 30 Hz for 8 min and the resulting powder was resuspended in 500 mL of water. To iron-saturate ferricrocin, the suspension was adjusted to 1 mM FeCl_3_, stirred for 30 min, and cleared of cellular debris by centrifugation at 2113 *g* for 10 min. The same XAD chromatography protocol described above was used to purify ferricrocin from the aqueous extract. Finally, the chloroform phase containing TAFC and the methanolic eluate containing ferricrocin were dried, and the siderophores were dissolved in distilled water for quantification and verification by HPLC. Ferrichrome was purchased from Sigma-Aldrich (USA) and coprogen B from EMC Microcollections (Germany).

### In silico identification of putative iron homeostatic proteins in lichen mycobionts, sequence alignments and phylogenetic analysis

Putative iron homeostatic proteins were identified using BLASTp-based homology searches and *A. fumigatus* query sequences were obtained from FungiDB (www.fungidb.org). For *X. parietina* mycobiont specifically, candidate proteins were identified by BLASTp searches against its annotated genome (*Xanthoria parietina* 46-1 v1.0, www.mycocosm.jgi.doe.gov/Xanpa1/Xanpa1.home.html). To broaden the analysis across lichens, homologs were retrieved by BLASTp against the NCBI non-redundant (nr) protein database^76^ and against species listed in Supplementary Data 1^77^. Selection criteria are given in Supplementary Data 1.

Alignments were made using Geneious Prime 2024.0.5 (www.geneious.com). The MOTIF Search website (www.genome.jp/tools/motif/) was used to analyze the motifs in the protein sequences. For statistical analyses, GraphPad Prism version 10.2.3 was used (GraphPad Software, USA; www.graphpad.com).

Maximum likelihood phylogenetic analysis of the identified *SidC* homologs from 41 lichen mycobiont species (see Supplementary Data 1) was performed including SidC homologs from 30 non-lichenized fungal species known to produce ferrichrome-type siderophores using the same selection criteria (Fig. 3c). All retained sequences were imported into Geneious Prime 2026.0.2. Sequences were aligned with MAFFT v7.490^78,79^ using FFT‑NS‑i x2 with BLOSUM62 scoring (gap‑open 1.53, offset 0.25), and alignment columns with ≥80% gaps were excluded. Maximum-likelihood phylogenies were inferred with RAxML v8.2.11^80^ using ‘Rapid bootstrapping and search for best-scoring ML tree’ (-f a). Searches were performed under LG with the CAT rate approximation (PROTCATLG) and the best tree was evaluated under Γ rate heterogeneity. Bootstrapping used the autoMRE stopping criterion with seeds -*p* 12345 and -*x* 67890 and converged after 100 replicates. The best‑scoring topology was then re‑optimized under LG with empirical amino‑acid frequencies and Γ‑distributed rates (PROTGAMMALGF; -f d), model parameters and branch lengths were refined on the fixed topology (-f e), and bootstrap bipartitions from the first run were mapped onto this final tree (-f b). Bootstrap support values are reported as percentages on the final unrooted tree.

### Expression analysis of *X. parietina* mycobiont genes

For expression analysis of the genes potentially involved in iron homeostasis of the *X. parietina* mycobiont, a recently published RNAseq dataset^41^ was re-analyzed, focusing on the genes listed in Table 1 and their differential expression in different environments. To identify the genes of interest in the genome from Tagirdzhanova et al. ^41^, we searched them against the predicted proteome using BLASTp. XpSidC retrieved three hits, resembling neighboring gene models because the gene has been split due to an assembly error that introduced a stop codon. In the downstream analysis, we used the gene model XANPAGTX0501_001694-T1, which covered the largest portion of the gene. We then ran differential expression analysis comparing samples collected on concrete and on tree bark. For this, a gene count matrix produced with kallisto version v0.46.2^81^ was used^41^. The matrix was then re-analyzed with sleuth v0.30.1^82^. We considered all genes with *q* value < 0.05 as differentially expressed.

### Reporting summary

Further information on research design is available in the Nature Portfolio Reporting Summary linked to this article.

## Supplementary information

Supplementary Information

Description of Additional Supplementary Files

Supplementary Data 1

Reporting Summary

Transparent Peer Review file

## Source data

Source Data

## Data Availability

All data are available in the main text, the Supplementary information or the Source Data file. The accession numbers of genes potentially involved in iron homeostasis are given in Table 1, the accession numbers of homologs of the *X. parietina* non-ribosomal ferrichrome peptide synthetase XpSidC in different lichen mycobionts are given in Supplementary Data 1, and the accession numbers of genes involved or potentially involved in iron acquisition in different microalgae are given in Table 2 and Supplementary Table S3. The transcriptome dataset used for extracting the data presented in Fig. 6 and Supplementary Table S1 is already public (CODE: https://github.com/metalichen/2024-Multipartite-complexity-omics-Xanthoria; PRJEB78723 and PRJEB38537)^41^. Source data are provided with this paper.
