# Supplementary material for: Siderophore production by the lichen fungus Xanthoria parietina supports its algal symbiont
Source: Nat Commun. 2026 Jul 2;17:8234. doi: 10.1038/s41467-026-74988-9 (PMC13462969; doi:10.1038/s41467-026-74988-9)
Supplement: Supplementary file 1 [file 41467_2026_74988_MOESM1_ESM.pdf]

Supplementary information for

Siderophore production by the lichen fungus *Xanthoria parietina* supports its algal symbiont

Isidor Happacher[1], Gregor Pichler[2,3], Beate Abt[1], Gulnara Tagirdzhanova[4,5], Simon Oberegger[1], Martin Kraihammer[6], Klaus Faserl[7], Bettina Sarg[7], Clemens Decristoforo[6], Roman Türk[8], Günther Winkelmann[9], Nicholas J. Talbot[4], Matthias Brock[10], Ilse Kranner[2], Kurt Haselwandter[11] and Hubertus Haas[1§]

This includes:

Supplementary Table S1. Differential expression of iron-associated genes in the *X. parietina* mycobiont. Genes of interest were identified in the genome of *X. parietina* GTX0501 isolate with BLASTp (query, percent identity, and E-value are provided). Only best hits and hits with E-value <1e-100 were considered. Differential expression analysis was performed using kallisto and sleuth. For the differentially expressed genes, q-value (corrected p-value) and b-value (analogous to logFC) are provided. For all genes, provide transcript per million (TPM) values in all samples is provided. Sample abbreviations: XBA, bark/apothecia; XBE, bark/thallus edge; XBC, bark/thallus center; XTA, twig/apothecia; XTC, twig/thallus center; XTE, twig/thallus edge; XSA, concrete/apothecia; XSC, concrete/thallus center; XSE, concrete/thallus edge; XMC, metal, thallus center

Supplementary Table S2. Ferrichrome content varies widely among *X. parietina* lichen samples from bark (n=3) and granite (n=3).

Supplementary Table S3. Homologous proteins involved or potentially involved in iron uptake in the microalgae *Trebouxia* sp., *Chlamydomonas reinhardtii, Dunaliella* and *Phaeodactylum tricornutum.* PSI-BLAST (Position-Specific Iterated BLAST) searches were conducted with 2 iterations in the NCBI database [1] limited to *Trebouxia* sp. (Taxonomy ID: 2796353, 13786, 285719), *Chlamydomonas reinhardtii* (Taxonomy ID: 3055), *Dunaliella* (Taxonomy ID: 3044), and *Phaeodactylum tricornutum* (Taxonomy ID: 2850). The microalgae species are highlighted in different colors. Query proteins are given in the headers of the individual Tables.

Supplementary Table S4. Raw data for PAM chlorophyll fluorescence analysis (F_v/F_m) of *T. decolorans* grown with 0.1 mM BPS, measured after 45 days of incubation and dark adaptation; values represent biological triplicates (n=3).

Supplementary Table S5. *Aspergillus fumigatus* strains and *Xanthoria parietina* samples used in this study

Supplementary Fig. S1. Fungal siderophore biosynthetic pathway. The first committed step in biosynthesis of hydroxamate-type siderophores is synthesis of N^5-hydroxy-L-ornithine from L-ornithine catalyzed by the monooxygenase SidA [4,5]. Subsequently, the pathways for the synthesis of fusarinine-type/coprogen-type and ferrichrome-type siderophores split due to incorporation of different acyl-groups. Synthesis of fusarinine-type and coprogen type siderophores involves the mevalonyl-coenzyme A (CoA) ligase SidI, the mevalonyl-CoA hydratase SidH, the transacylase SidF, and the non-ribosomal peptide synthetases (NRPS) SidD [6]. Biosynthesis of TAFC additionally requires the transacetylase SidG [4,7,8]. Ferrichrome-type siderophore synthesis involves the transacetylases SidL and SidF, and the NRPS SidC [8–11]. SidI, SidH and SidF are localized in peroxisomes, whereas the other enzymes are thought to be found in the cytoplasm [12]. Siderophores and siderophore pathway-specific intermediates are written in **bold**.

Supplementary Fig. S2. Maximum likelihood phylogenetic analysis of the identified *SidC* homologues from 41 lichen mycobiont species and 30 non-lichenized fungal species known to produce ferrichrome-type siderophores. Mycobiont SidC homologues are listed in Supplementary Data 1. Sequences were retained from BLASTp hits with E-value ≤ 5.92e-100 and protein length ≥ 2,000 amino acids. The clade containing functionally characterized SidC homologs (shaded light grey here) is shown in Fig. 3c. Bootstrap support (%) of this unrooted tree is indicated at nodes. No characterized SidC homologs occur outside this clade, indicating that it encompasses the NRPSs that produce ferrichrome-type siderophores. Proteins discussed in Fig. 3b are highlighted in **bold**.

Supplementary Fig. S3. Amino acid alignment of *X. parietina* XpSidC and *A. fumigatus* SidC. A Clustal Omega alignment of the protein sequences was performed using Geneious Prime. The annotations of the sequences were obtained from the MOTIF Search website (https://www.genome.jp/tools/motif/): adenylation domain in purple, thiolation site in black and condensation domain in blue. Identical amino acid residues are indicated in green.

Supplementary Fig. S4. SidC homologs from analyzed OSLEUM clade lichen mycobionts share a compact NRPS architecture, apart from *Peltigerales* species. A Clustal Omega alignment of the protein sequences was performed using Geneious Prime. The annotations of the sequences were obtained from the MOTIF Search website (https://www.genome.jp/tools/motif/): adenylation domain in purple, thiolation domain in black, condensation domain in blue and L-ornithine 5-monooxygenase domain in gold. Sequences from *Lecanoromycetidae* are shown in violet, *Ostropomycetidae* in turquoise, non-OSLEUM clade (*Chaetothyriomycetidae*, *Dothideomycetes*, and *Leotiomyceta*) in light red, and non-lichenized species in black. Proteins discussed in Fig. 3b are highlighted in bold. Presence of an L-ornithine 5-monooxygenase domain may indicate fusion proteins or incorrect annotation. The unusual short sequences from *Bacidia gigantensis*, *Usnochroma carphineum*, *Peltigera leucophlebia* and *Variospora aurantia* indicate incorrect annotation.

Supplementary Fig. S5. Schemes (a) and Southern blot conformation (b) of the genetic manipulations at the *fcyB* locus for the integration of *X. parietina* XpSidC in *A. fumigatus*. Restriction enzyme digested genomic DNA was subjected to Southern blot analysis. *Hind*III digestion resulted in a fragment length of 2,727 bp in the recipient strain Δ*sidC* and 8,314 bp in Δ$sidC^{XpSidC}$. Primers IH141 (TGCGGTTTTTGGGTTTTATCAATTGG) and IH142 (GCGGTATGAAACAACGGTCT) were used to amplify the DIG probe. The results are in line with the *in silico* predictions. (**c**) Uncropped Southern blot image (conducted once).

Supplementary information References

Supplementary Table S1. Differential expression of iron-associated genes in the *X. parietina* mycobiont. Genes of interest were identified in the genome of *X. parietina* GTX0501 isolate with BLASTp (query, percent identity, and E-value are provided). Only best hits and hits with E-value <1e-100 were considered. Differential expression analysis was performed using kallisto and sleuth. For the differentially expressed genes, q-value (corrected p-value) and b-value (analogous to logFC) are provided. For all genes, provide transcript per million (TPM) values in all samples is provided. Sample abbreviations: XBA, bark/apothecia; XBE, bark/thallus edge; XBC, bark/thallus center; XTA, twig/apothecia; XTC, twig/thallus center; XTE, twig/thallus edge; XSA, concrete/apothecia; XSC, concrete/thallus center; XSE, concrete/thallus edge; XMC, metal, thallus center

gene	**Query** from *Xanthoria parietina* 46-1 v1.0 JGI genome	**Transcript ID** from *X. parietina* GTX0501 genome	InterProScan annotation	percent identical	E-value	differential expression	q-value	b-value
**SreA**	11759 | fgenesh1_pg.10_#_325	XANPAGTX0501_004153-T1	IPR000679; IPR013088	99.828	0	not differentially expressed		
**HapX**	11971 | fgenesh1_pg.11_#_162	XANPAGTX0501_009508-T1	IPR004827; IPR018287	98.319	0	not differentially expressed		
**SidA**	14819 | fgenesh1_pg.24_#_33	XANPAGTX0501_001696-T1	IPR025700; IPR036188	99.336	0	not differentially expressed		
**CccA**	53727 | e_gw1.20.104.1	XANPAGTX0501_010113-T1	IPR008217	99.083	0	not differentially expressed		
**SidC**	54414 | e_gw1.24.24.1	XANPAGTX0501_001694-T1	IPR000873; IPR001242; IPR006162; IPR009081; IPR020845; IPR023213; IPR036736; IPR042099	99.013	0	up-regulated in concrete	0.01670	0.44905
**FetC**	55657 | fgenesh1_pm.2_#_237	XANPAGTX0501_000369-T1	IPR001117; IPR002355; IPR008972; IPR011706; IPR011707; IPR033138	98.801	0	not differentially expressed		
**FtrA**	55658 | fgenesh1_pm.2_#_238	XANPAGTX0501_000371-T1	IPR004923	99.183	0	not differentially expressed		
**SidL**	57462 | fgenesh1_pm.7_#_41	XANPAGTX0501_006174-T1	IPR016181; IPR019432	99.055	0	up-regulated in bark	0.00009	-0.36246
**SidT**	60333 | fgenesh1_pm.24_#_13	XANPAGTX0501_001695-T1	IPR003439; IPR003593; IPR011527; IPR017871; IPR027417; IPR036640	93.388	0	up-regulated in concrete	0.01514	0.35974
**Sit2**	60974 | gm1.629_g	XANPAGTX0501_009320-T1	IPR011701; IPR020846; IPR036259	99.485	0	up-regulated in concrete	0.00551	0.58678
**MirC**	70210 | gm1.9865_g	XANPAGTX0501_006567-T1	IPR011701; IPR020846; IPR036259	99.670	0	up-regulated in concrete	0.00057	0.31415

**lichen collected from:**	**bark**										**concrete**						**metal**
**gene**	**XBE1**	**XBC1**	**XBA1**	**XBE2**	**XBC2**	**XBA2**	**XTA1**	**XTA2**	**XTE2**	**XTC2**	**XSC1**	**XSA1**	**XSE2**	**XSC2**	**XSA2**	**XSA2_2**	**XMC2**
**SreA**	15.14690	13.96720	12.91680	12.82840	16.66130	14.16940	14.85620	16.16520	13.36740	16.48060	12.45820	13.23280	19.88700	17.76610	15.23320	18.94800	21.38490
**HapX**	6.07987	5.79493	8.94452	5.31875	7.51881	7.83187	8.29228	9.36942	8.04937	8.96845	4.31430	5.57247	7.48265	7.58673	6.81095	8.06063	5.82526
**SidA**	7.50163	5.57501	5.33977	9.90484	6.28799	6.82541	6.90254	7.50760	6.14954	5.81217	9.54542	10.29940	9.19003	9.33364	7.36768	8.65255	9.21262
**CccA**	38.73870	27.90560	29.33600	32.36130	27.63340	26.04440	29.31520	29.07150	30.51820	32.54040	26.05750	18.98390	54.07760	42.49520	47.92180	42.32170	27.90160
**SidC**	0.89202	0.67659	0.63929	0.63660	1.35530	1.31453	1.53664	1.41527	0.63744	1.20009	1.54931	1.71570	2.06914	1.86388	0.83498	2.07935	1.33872
**FetC**	24.95680	18.69480	17.45060	18.82600	17.18020	14.11190	20.33870	19.38020	17.24300	19.53230	21.66740	20.69330	30.07280	27.02280	18.51170	25.00530	30.12780
**FtrA**	26.01420	23.92950	25.27600	24.11820	16.72800	14.37480	26.34120	22.89940	18.58200	21.31360	20.55130	20.12850	32.23910	29.70430	19.05950	26.95530	29.26410
**SidL**	38.37650	44.52760	35.51230	28.08540	28.60070	29.26720	31.77530	38.15780	31.78350	36.20180	19.82400	21.13150	30.17610	30.88490	22.73030	27.66400	30.58080
**SidT**	2.14446	1.82942	1.45777	1.83434	1.89698	1.45672	2.99712	2.38730	2.02830	2.40549	3.29343	3.49810	3.54078	3.60859	1.68454	3.21700	3.07025
**Sit2**	1.70153	1.35559	2.06802	1.73133	2.06066	2.05871	0.67959	3.30020	1.24372	2.43894	2.29671	2.70955	3.85866	4.33072	3.22561	3.91529	3.22231
**MirC**	26.13430	26.82060	30.87330	24.58260	29.99580	28.60850	30.36080	31.29010	25.09630	30.49110	27.51470	31.25690	51.03640	54.73250	40.51790	50.07240	31.53180

Supplementary Table S2. Ferrichrome content varies widely among *X. parietina* lichen samples from bark (n=3) and granite (n=3).

	Sample	Ferrichrome [pmol/mg]	Mean [pmol/mg]
**bark**	A	4.913	3.035
	B	1.837	
	C	2.354	
**granite**	D	8.448	5.586
	E	4.949	
	F	3.360	

Supplementary Table S3. Homologous proteins involved or potentially involved in iron uptake in the microalgae *Trebouxia* sp., *Chlamydomonas reinhardtii, Dunaliella* and *Phaeodactylum tricornutum.* PSI-BLAST (Position-Specific Iterated BLAST) searches were conducted with 2 iterations in the NCBI database [1] limited to *Trebouxia* sp. (Taxonomy ID: 2796353, 13786, 285719), *Chlamydomonas reinhardtii* (Taxonomy ID: 3055), *Dunaliella* (Taxonomy ID: 3044), and *Phaeodactylum tricornutum* (Taxonomy ID: 2850). The microalgae species are highlighted in different colors. Query proteins are given in the headers of the individual Tables.

Query: ***Aspergillus fumigatus* metalloreductase FreB**; Q4WR75.2; 743 amino acids

Scientific Name	Max Score	Total Score	Query Cover (%)	E value	Per. Ident.	Acc. Len.	Accession
Trebouxia sp. C0006	347	347	62 %	6.00E-108	22.82	748	KAL0043219.1
Trebouxia sp. C0006	343	343	62 %	3.00E-106	22.82	765	KAL0043220.1
Trebouxia sp. C0009 RCD-2024	338	338	59 %	5.00E-104	19.31	799	KAL3146993.1
Trebouxia sp. C0010 RCD-2024	335	335	59 %	2.00E-102	19.52	815	KAL3134756.1
Chlamydomonas reinhardtii	316	316	48 %	2.00E-93	20.98	1087	XP_042925384.1
Dunaliella salina	296	296	56 %	2.00E-86	19.19	1038	KAF5840498.1
Trebouxia sp. C0004	267	267	57 %	9.00E-78	16.82	755	KAL0041029.1
Trebouxia sp. C0005	260	260	57 %	1.00E-75	16.82	721	KAL0043585.1
Trebouxia sp. A1-2	258	258	57 %	2.00E-74	16.82	813	KAA6424729.1
Trebouxia sp. C0006	253	253	57 %	2.00E-72	17.26	813	KAL0045657.1
Dunaliella salina	227	227	55 %	1.00E-61	17.98	1213	KAF5833427.1
Trebouxia sp. C0010 RCD-2024	183	183	72 %	4.00E-48	17.6	691	KAL3136132.1
Trebouxia sp. C0009 RCD-2024	182	182	59 %	1.00E-47	17.49	746	KAL3151709.1
Trebouxia sp. C0010 RCD-2024	182	182	68 %	2.00E-47	16.91	847	KAL3159196.1
Phaeodactylum tricornutum CCAP 1055/1	179	179	29 %	6.00E-47	23.75	656	XP_002185965.1
Trebouxia sp. C0005	180	180	59 %	7.00E-47	17.71	755	KAL0040033.1
Trebouxia sp. C0010 RCD-2024	179	179	68 %	9.00E-47	16.91	737	KAL3159197.1
Trebouxia sp. C0004	173	173	64 %	2.00E-44	16.47	795	KAL0029044.1
Chlamydomonas reinhardtii	164	164	51 %	4.00E-42	16.49	620	XP_042926276.1
Chlamydomonas reinhardtii	150	150	52 %	1.00E-37	17.99	555	XP_042926274.1
Phaeodactylum tricornutum CCAP 1055/1	109	109	56 %	1.00E-23	15.62	1326	XP_002180058.1
Trebouxia sp. C0009 RCD-2024	92.6	92.6	58 %	2.00E-18	14.59	801	KAL3151900.1
Phaeodactylum tricornutum CCAP 1055/1	86.8	86.8	52 %	9.00E-17	17.63	746	XP_002179812.1
Chlamydomonas reinhardtii	87.2	87.2	46 %	1.00E-16	14.86	1139	XP_042924943.1
Phaeodactylum tricornutum CCAP 1055/1	78.7	78.7	45 %	3.00E-14	16.71	746	XP_002183592.1
Trebouxia sp. C0009 RCD-2024	71	71	33 %	6.00E-12	16.99	489	KAL3131593.1
Trebouxia sp. C0010 RCD-2024	66.4	66.4	46 %	2.00E-10	15.96	484	KAL3159009.1
Trebouxia sp. A1-2	65.2	65.2	17 %	4.00E-10	20.61	704	KAA6420141.1
Trebouxia sp. C0005	64.8	108	36 %	6.00E-10	20.61	730	KAL0027981.1
Trebouxia sp. C0004	64	64	32 %	1.00E-09	16.07	826	KAL0020140.1
Trebouxia sp. C0006	63.7	114	36 %	1.00E-09	22.14	825	KAL0054753.1

Query: ***Aspergillus fumigatus* ferroxidase FetC;** E9R598.1; 592 amino acids

Scientific Name	Max Score	Total Score	Query Cover (%)	E value	Per. Ident.	Acc. Len.	Accession
Chlamydomonas reinhardtii	470	470	81 %	1.00E-156	21.64	777	XP_042927303.1
Trebouxia sp. C0004	457	457	83 %	4.00E-152	24.32	751	KAL0024990.1
Chlamydomonas reinhardtii	373	373	81 %	7.00E-120	23.55	719	XP_042928556.1
Trebouxia sp. C0009 RCD-2024	334	334	53 %	2.00E-106	25.95	571	KAL3136665.1
Trebouxia sp. C0005	335	484	70 %	1.00E-105	26.84	691	KAL0038686.1
Trebouxia sp. C0006	332	478	67 %	7.00E-104	27.74	752	KAL0043919.1
Trebouxia sp. C0010 RCD-2024	313	313	43 %	3.00E-101	25	374	KAL3145498.1
Chlamydomonas reinhardtii	153	304	43 %	2.00E-38	39.82	1102	XP_042925853.1
Trebouxia sp. A1-2	144	144	23 %	3.00E-37	32.48	374	KAA6419185.1
Trebouxia sp. C0009 RCD-2024	136	136	18 %	6.00E-37	37.38	154	KAL3136666.1
Trebouxia sp. C0010 RCD-2024	138	138	18 %	2.00E-35	38.32	358	KAL3145497.1
Chlamydomonas reinhardtii	119	254	48 %	3.00E-27	26.85	1233	XP_042918663.1
Chlamydomonas reinhardtii	114	247	79 %	1.00E-25	27.85	1142	XP_001694585.2
Trebouxia sp. A1-2	99.1	99.1	15 %	1.00E-22	17.88	235	KAA6419193.1
Dunaliella salina	90.2	188	80 %	5.00E-18	25.71	1006	KAF5833657.1
Dunaliella salina	89.8	186	80 %	9.00E-18	25.71	1233	KAF5833656.1
Dunaliella salina	75.9	168	49 %	2.00E-13	21.09	1075	AAX88807.1
Chlamydomonas reinhardtii	71.7	71.7	82 %	4.00E-12	19.49	1377	XP_042922759.1
Chlamydomonas reinhardtii	59.4	59.4	89 %	2.00E-08	17.68	1318	XP_042922775.1
Trebouxia sp. C0009 RCD-2024	52.1	52.1	56 %	4.00E-06	18.16	622	KAL3147414.1
Trebouxia sp. C0009 RCD-2024	51.3	51.3	56 %	6.00E-06	18.16	601	KAL3147415.1
Trebouxia sp. C0009 RCD-2024	51.3	51.3	56 %	7.00E-06	18.16	710	KAL3147413.1
Trebouxia sp. C0010 RCD-2024	50.5	50.5	56 %	1.00E-05	19.38	558	KAL3162130.1
Trebouxia sp. C0010 RCD-2024	50.5	50.5	56 %	1.00E-05	19.38	710	KAL3162129.1
Trebouxia sp. C0005	48.2	48.2	49 %	7.00E-05	17.82	722	KAL0033599.1
Trebouxia sp. C0004	48.2	48.2	56 %	8.00E-05	17.43	1466	KAL0039081.1
Trebouxia lynnae	47.1	47.1	56 %	2.00E-04	17.63	718	QOL01226.1
Trebouxia sp. C0006	46.7	46.7	56 %	2.00E-04	17.29	710	KAL0046873.1
Trebouxia sp. C0004	45.1	45.1	67 %	5.00E-04	18.16	657	KAL0039232.1
Trebouxia sp. C0005	43.6	43.6	56 %	0.002	17.39	711	KAL0022272.1

Query: ***Aspergillus fumigatus* high-affinity iron transporter FtrA;** XP_747964.2; 370 amino acids

Scientific Name	Max Score	Total Score	Query Cover (%)	E value	Per. Ident.	Acc. Len.	Accession
Trebouxia sp. C0006	433	433	96 %	2.00E-148	30.85	584	KAL0047096.1
Trebouxia sp. C0004	406	406	98 %	2.00E-140	31.06	390	KAL0029032.1
Trebouxia sp. C0010 RCD-2024	392	392	92 %	5.00E-135	32.26	392	KAL3141519.1
Trebouxia sp. C0005	371	371	89 %	9.00E-128	31.42	324	KAL0018724.1
Trebouxia sp. C0009 RCD-2024	373	373	92 %	2.00E-127	31.87	392	KAL3155144.1
Trebouxia sp. A1-2	292	345	86 %	1.00E-96	30.55	313	KAA6417637.1
Chlamydomonas reinhardtii	197	349	86 %	2.00E-57	26.42	541	XP_001693245.1
Trebouxia sp. A1-2	164	164	38 %	1.00E-48	25.17	186	KAA6416631.1

Query: ***Phaeodactylum tricornutum* ISIP1;** XP_002183904.1; 569 amino acids

Scientific Name	Max Score	Total Score	Query Cover (%)	E value	Per. Ident.	Acc. Len.	Accession
Phaeodactylum tricornutum CCAP 1055/1	833	833	100 %	0	100	569	XP_002183904.1
Dunaliella salina	422	649	79 %	1.00E-135	21.88	1054	AAX88808.2

Dunaliella salina	416	637	79 %	4.00E-133	22.45	1071	KAF5842622.1
Dunaliella salina	379	379	77 %	4.00E-124	15.72	611	KAF5839271.1
Trebouxia lynnae	258	258	53 %	3.00E-77	16.72	634	QOL01286.1
Trebouxia sp. C0010 RCD-2024	242	242	38 %	3.00E-72	16.74	540	KAL3150830.1
Trebouxia sp. C0004	230	230	39 %	7.00E-68	18.91	529	KAL0017780.1
Trebouxia sp. C0010 RCD-2024	222	222	35 %	5.00E-65	15.98	499	KAL3150831.1
Trebouxia sp. C0004	203	203	37 %	2.00E-57	20.25	553	KAL0017775.1
Trebouxia sp. C0004	201	201	37 %	9.00E-57	18.14	553	KAL0018562.1
Trebouxia sp. C0004	195	195	28 %	1.00E-54	15.79	525	KAL0018558.1
Trebouxia sp. C0009 RCD-2024	190	190	34 %	2.00E-53	17.73	485	KAL3137606.1
Trebouxia sp. C0004	193	193	37 %	8.00E-52	14.85	1033	KAL0035545.1

Query: ***Dunaliella salina* transferrin-like protein TTF-1;** AAB36531.1; 1274 amino acids

Scientific Name	Max Score	Total Score	Query Cover (%)	E value	Per. Ident.	Acc. Len.	Accession
Dunaliella salina	1686	1686	99 %	0	100	1274	AAB36531.1
Dunaliella tertiolecta	1629	1629	99 %	0	97.16	1274	AAF72064.1
Dunaliella salina	1482	2722	100 %	0	71.97	1709	KAF5830738.1
Dunaliella salina	1039	2742	92 %	0	50.05	1534	KAF5834923.1
Dunaliella salina	928	2093	97 %	0	42.57	1558	KAF5834924.1
Dunaliella salina	781	1538	96 %	0	29.65	972	KAF5841150.1
Dunaliella salina	647	1182	99 %	0	26.94	908	AAN39804.1
Trebouxia sp. C0010 RCD-2024	601	1111	91 %	0	22.59	769	KAL3141978.1
Trebouxia sp. C0009 RCD-2024	590	1093	91 %	0	22.8	767	KAL3153465.1
Dunaliella salina	525	1197	93 %	5.00E-172	27.54	602	KAF5827187.1
Trebouxia sp. C0010 RCD-2024	412	1027	91 %	1.00E-129	20.9	560	KAL3141979.1
Dunaliella salina	320	950	91 %	7.00E-97	27.29	477	KAF5830737.1
Dunaliella salina	284	707	62 %	6.00E-86	47.29	322	KAF5843732.1
Dunaliella salina	186	580	39 %	4.00E-51	64.44	353	KAF5843731.1
Dunaliella salina	164	421	30 %	4.00E-46	70.08	135	KAF5825248.1
Dunaliella salina	89.2	89.2	7 %	3.00E-20	45.24	105	KAF5825609.1

Query: ***Phaeodactylum tricornutum* ferrichrome-binding protein FBP1;** XP_002185795.1; 300 amino acids

Scientific Name	Max Score	Total Score	Query Cover (%)	E value	Per. Ident.	Acc. Len.	Accession
Phaeodactylum tricornutum CCAP 1055/1	617	617	100 %	0	100	300	XP_002185795.1

Supplementary Table S4. Raw data for PAM chlorophyll fluorescence analysis (F_v/F_m) of *T. decolorans* grown with 0.1 mM BPS, measured after 45 days of incubation and dark adaptation; values represent biological triplicates (n=3).

	F_v/F_m		
**-**	0	0	0
**iron-free ferrichrome**	0.183	0.167	0.236
**$FeSO_4$**	0	0	0
**ferrichrome-iron chelate**	0.308	0.335	0.365

Supplementary Table S5. *Aspergillus fumigatus* strains and *Xanthoria parietina* samples used in this study

***A. fumigatus* strains**			
**Strain-name**	**Genotype**	**Reference**	
wt (AfS77 wild type)	ATCC46645; Δ*akuA::loxP*	[2]	
Δ*sidA*Δ*ftrA*	AfS77; Δ*sidA::six*, Δ*ftrA::six*	[3]	
Δ*sidA*Δ*ftrA*Δ*sit1*	AfS77; Δ*sidA::six*, Δ*ftrA::six*, Δ*sit1::hph*	[3]	
Δ*sidA*Δ*ftrA*Δ*sit2*	AfS77; Δ*sidA::six*, Δ*ftrA::six*, Δ*sit2::ptrA*	[3]	
Δ*sidA*Δ*ftrA*Δ*sit1*Δ*sit2*	AfS77; Δ*sidA::six*, Δ*ftrA::six*, Δ*sit1::hph*, Δ*sit2::ptrA*	[3]	
Δ*sidC*	ATCC46645; Δ*sidC::hph*	[4]	
Δ*sidC*^{XpSidC}	ATCC46645; Δ*sidC::hph*, Δ*fcyB::*P*xylP:XpSidC*	this study	
***X. parietina* collections from Austria**			
**Substrate**	**Location**	**Title**	**Notes**
bark<br>*Salix* sp.	Austria, Salzburg, Flachgau, Seekirchen, 511 msm.<br>N 47° 53‘ 42‘‘ E 013° 08‘ 09‘‘	Collected by R. Türk on 08.03.2023	The sample was used for initial identification of the siderophore.
bark<br>*Acer pseudoplatanus*	Austria, Upper Austria, Schärding, Kopfing, 540 msm.<br>N 48° 26' 14" E 013° 39' 30"	Collected by F. Berger on 21.01.2026	These samples were used to quantify ferrichrome in bark versus granite.
bark<br>*Acer pseudoplatanus*	Austria, Upper Austria, Schärding, Kopfing, 540 msm.<br>N 48° 26' 14" E 013° 39' 28"	Collected by F. Berger on 21.01.2026	
bark<br>*Acer pseudoplatanus*	Austria, Upper Austria, Schärding, Kopfing, 540 msm.<br>N 48° 26' 14" E 013° 39' 32"	Collected by F. Berger on 21.01.2026	
granite	Austria, Upper Austria, Schärding, St. Aegidi, 579 msm.<br>N 48° 26' 54" E 013° 42' 09"	Collected by F. Berger on 21.01.2026	
granite	Austria, Upper Austria, Kirchdorf, Scharnstein, 545 msm.<br>N 47° 53' 49.5" E 013° 56' 36"	Collected by F. Berger on 03.02.2026	
granite	Austria, Upper Austria, Sauwald, St. Agidi, 600 msm.<br>N 48° 26' 54" E 013° 42' 09"	Collected by F. Berger on 08.01.2026	

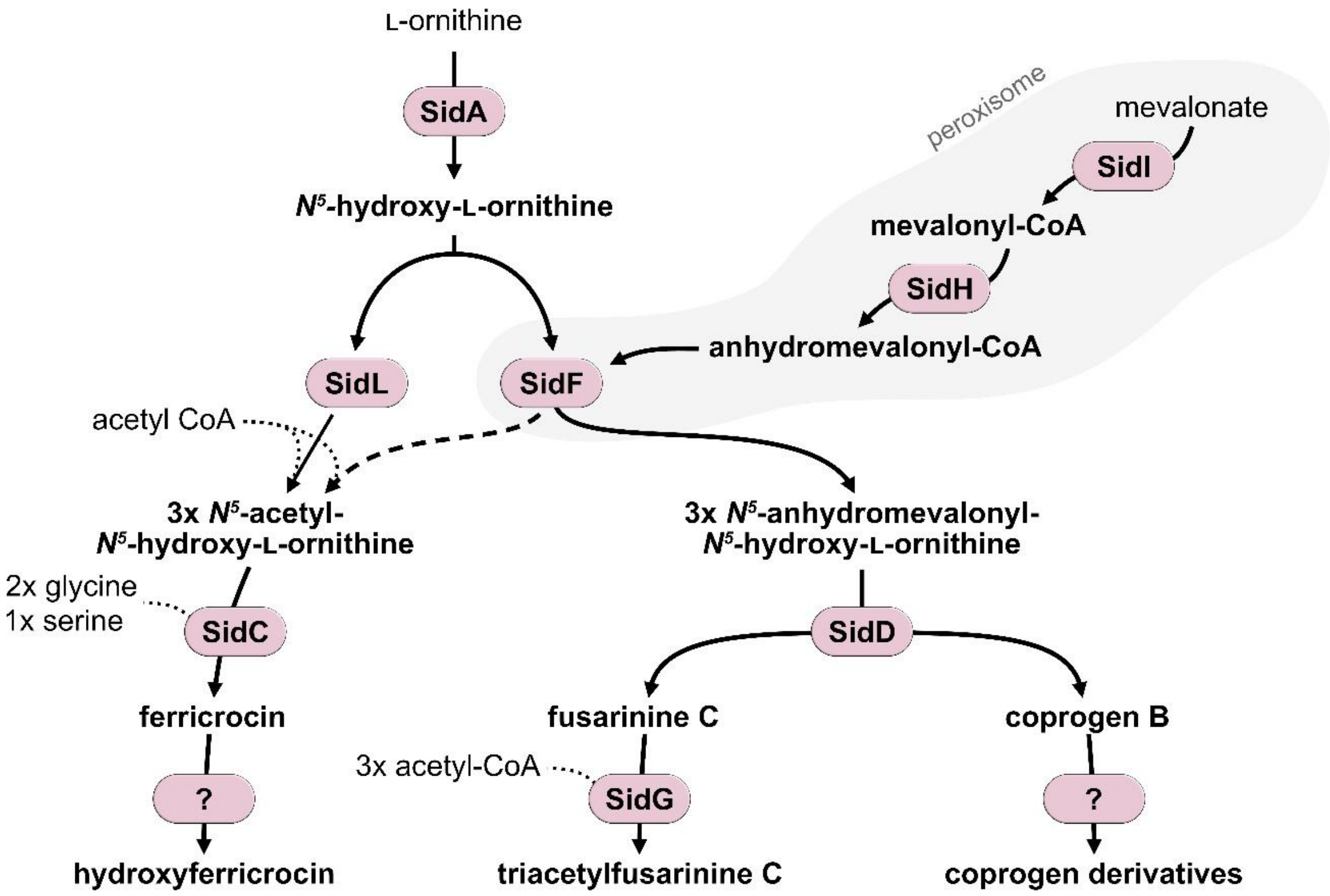

Supplementary Fig. S1. Fungal siderophore biosynthetic pathway. The first committed step in biosynthesis of hydroxamate-type siderophores is synthesis of N^5-hydroxy-L-ornithine from L-ornithine catalyzed by the monooxygenase SidA [4,5]. Subsequently, the pathways for the synthesis of fusarinine-type/coprogen-type and ferrichrome-type siderophores split due to incorporation of different acyl-groups. Synthesis of fusarinine-type and coprogen type siderophores involves the mevalonyl-coenzyme A (CoA) ligase SidI, the mevalonyl-CoA hydratase SidH, the transacylase SidF, and the non-ribosomal peptide synthetases (NRPS) SidD [6]. Biosynthesis of TAFC additionally requires the transacetylase SidG [4,7,8]. Ferrichrome-type siderophore synthesis involves the transacetylases SidL and SidF, and the NRPS SidC [8–11]. SidI, SidH and SidF are localized in peroxisomes, whereas the other enzymes are thought to be found in the cytoplasm [12]. Siderophores and siderophore pathway-specific intermediates are written in **bold**.

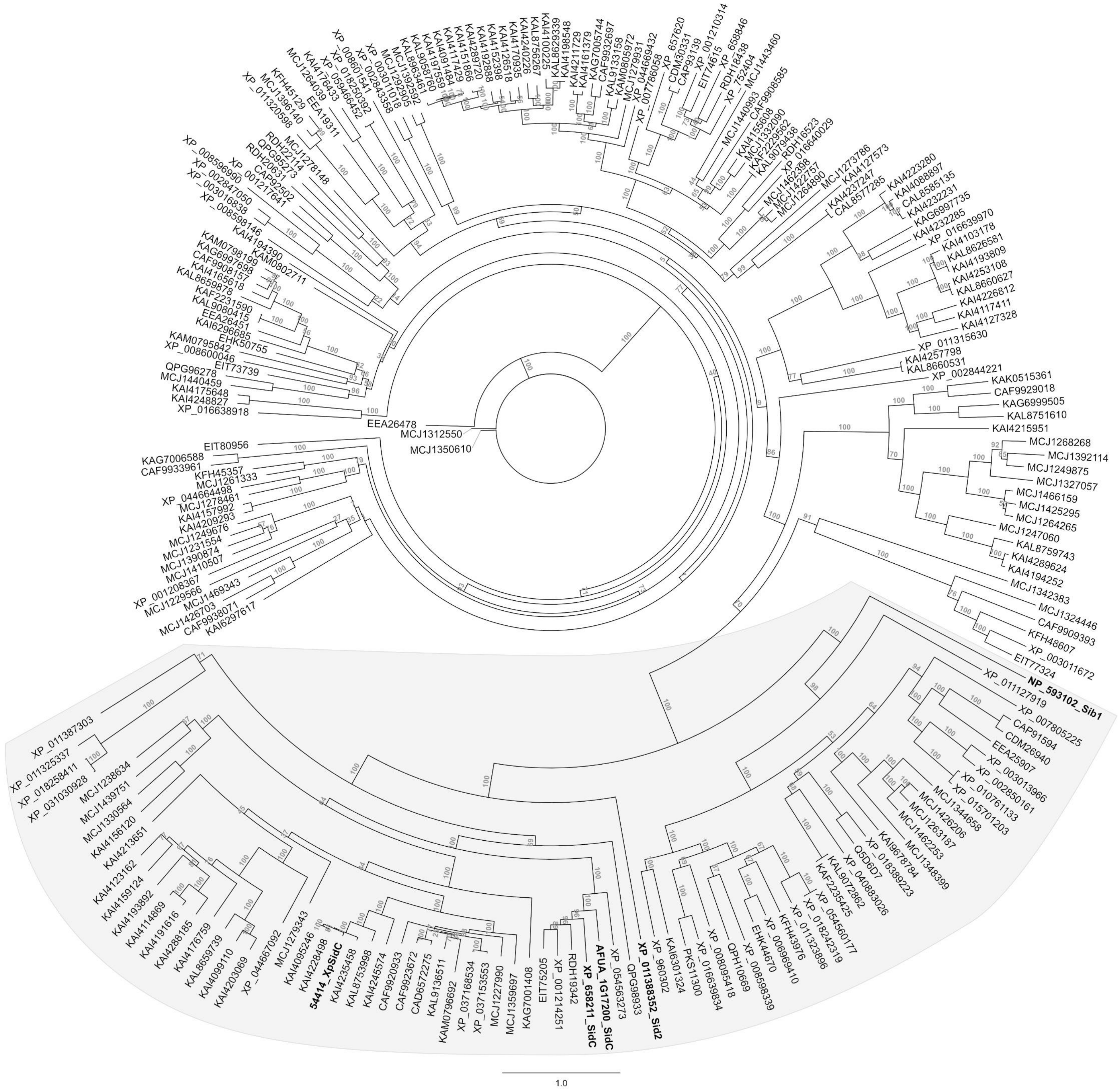

Supplementary Fig. S2. Maximum likelihood phylogenetic analysis of the identified *SidC* homologues from 41 lichen mycobiont species and 30 non-lichenized fungal species known to produce ferrichrome-type siderophores. Mycobiont SidC homologues are listed in Supplementary Data 1. Sequences were retained from BLASTp hits with E-value ≤ 5.92e-100 and protein length ≥ 2,000 amino acids. The clade containing functionally characterized SidC homologs (shaded light grey here) is shown in Fig. 3c. Bootstrap support (%) of this unrooted tree is indicated at nodes. No characterized SidC homologs occur outside this clade, indicating that it encompasses the NRPSs that produce ferrichrome-type siderophores. Proteins discussed in Fig. 3b are highlighted in **bold**.

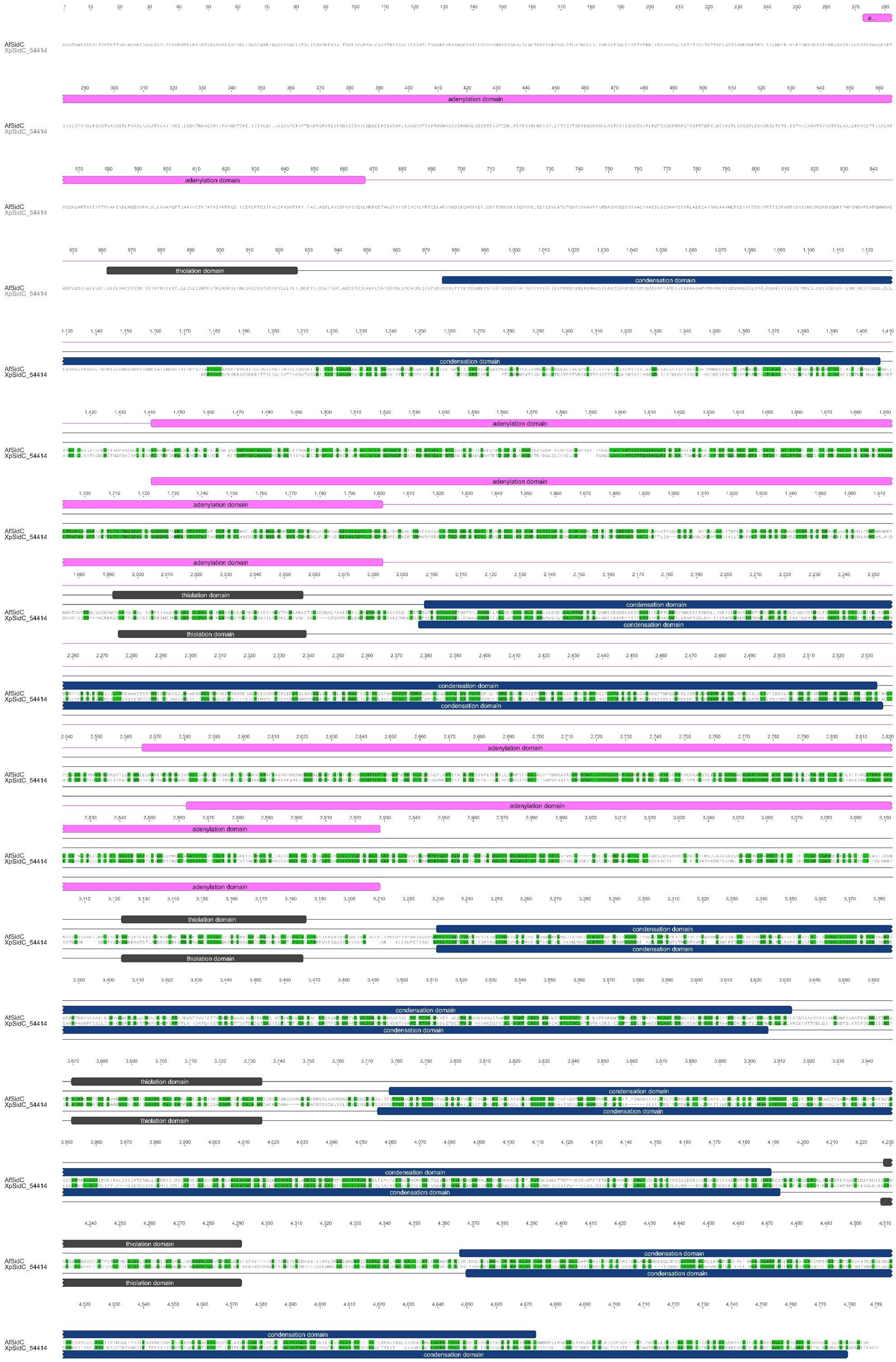

Supplementary Fig. S3. Amino acid alignment of *X. parietina* XpSidC and *A. fumigatus* SidC. A Clustal Omega alignment of the protein sequences was performed using Geneious Prime. The annotations of the sequences were obtained from the MOTIF Search website (https://www.genome.jp/tools/motif/): adenylation domain in purple, thiolation site in black and condensation domain in blue. Identical amino acid residues are indicated in green.

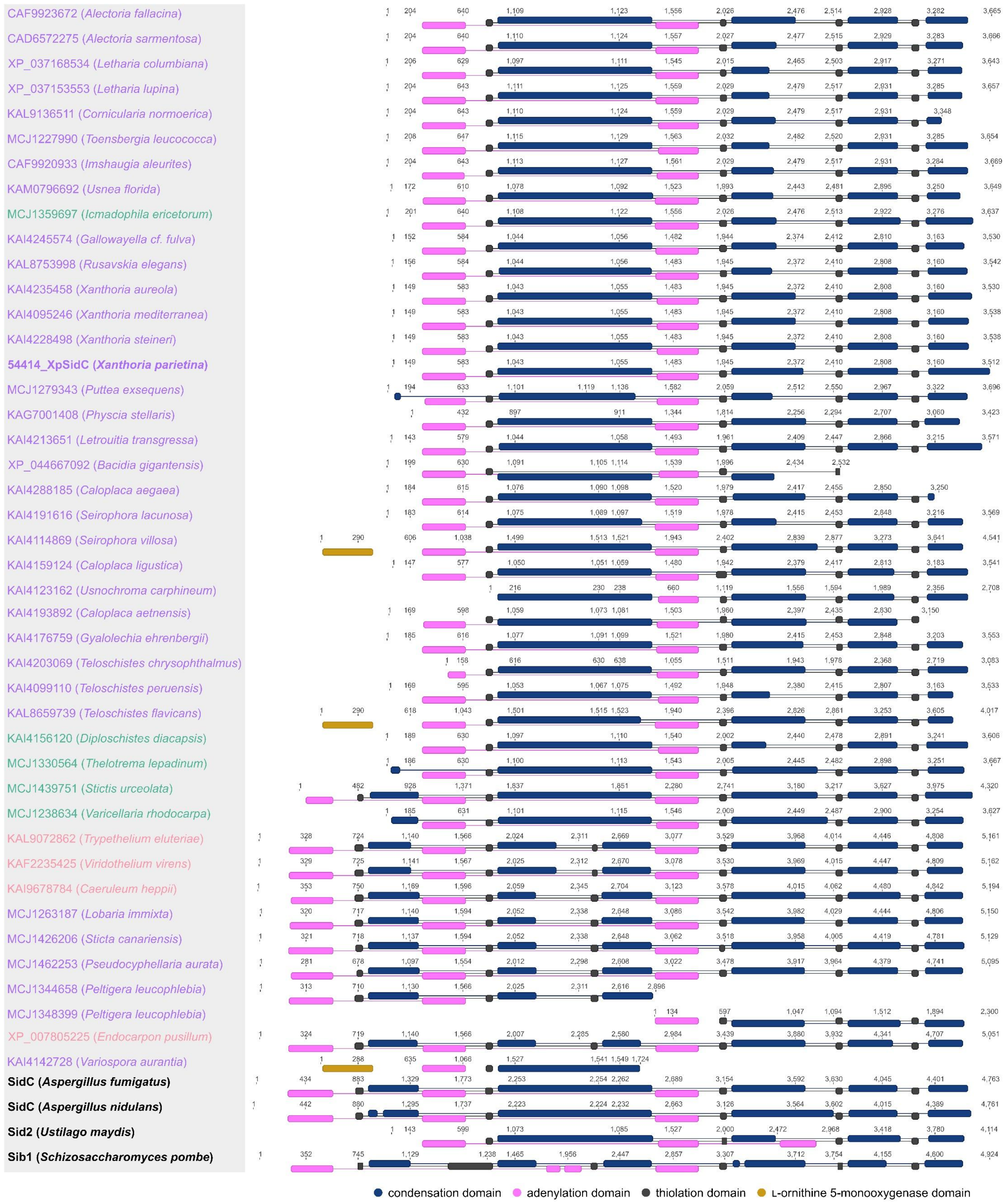

Supplementary Fig. S4. SidC homologs from analyzed OSLEUM clade lichen mycobionts share a compact NRPS architecture, apart from *Peltigerales* species. A Clustal Omega alignment of the protein sequences was performed using Geneious Prime. The annotations of the sequences were obtained from the MOTIF Search website (https://www.genome.jp/tools/motif/): adenylation domain in purple, thiolation domain in black, condensation domain in blue and L-ornithine 5-monooxygenase domain in gold. Sequences from *Lecanoromycetidae* are shown in violet, *Ostropomycetidae* in turquoise, non-OSLEUM clade (*Chaetothyriomycetidae*, *Dothideomycetes*, and *Leotiomyceta*) in light red, and non-lichenized species in black. Proteins discussed in Fig. 3b are highlighted in bold. Presence of an L-ornithine 5-monooxygenase domain may indicate fusion proteins or incorrect annotation. The unusual short sequences from *Bacidia gigantensis*, *Usnochroma carphineum*, *Peltigera leucophlebia* and *Variospora aurantia* indicate incorrect annotation.

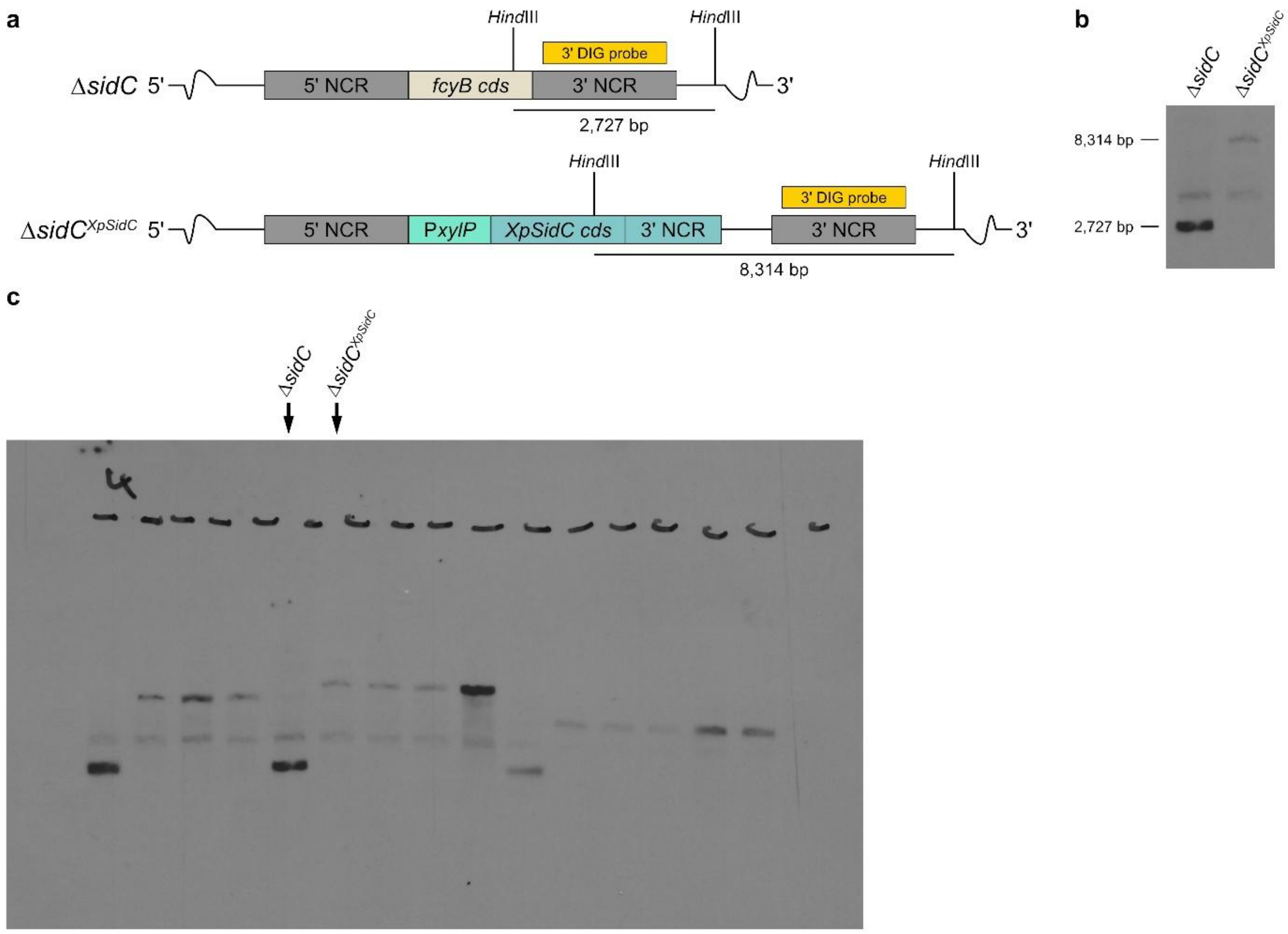

Supplementary Fig. S5. Schemes (a) and Southern blot conformation (b) of the genetic manipulations at the *fcyB* locus for the integration of *X. parietina* XpSidC in *A. fumigatus*. Restriction enzyme digested genomic DNA was subjected to Southern blot analysis. *Hind*III digestion resulted in a fragment length of 2,727 bp in the recipient strain Δ*sidC* and 8,314 bp in Δ$sidC^{XpSidC}$. Primers IH141 (TGCGGTTTTTGGGTTTTATCAATTGG) and IH142 (GCGGTATGAAACAACGGTCT) were used to amplify the DIG probe. The results are in line with the *in silico* predictions. (**c**) Uncropped Southern blot image (conducted once).

Supplementary References

1. Sayers, E. W. *et al.* Database resources of the National Center for Biotechnology Information. *Nucleic Acids Research* **47**, D23–D28 (2019).
2. Hartmann, T. *et al*. Validation of a self-excising marker in the human pathogen *Aspergillus fumigatus* by employing the beta-rec/six site-specific recombination system. *Appl. Environ. Microbiol*. **76**, 6313–6317 (2010).
3. Aguiar, M. *et al.* The Siderophore Transporters Sit1 and Sit2 Are Essential for Utilization of Ferrichrome-, Ferrioxamine- and Coprogen-Type Siderophores in *Aspergillus fumigatus*. *J Fungi (Basel)* **7**, 768 (2021).
4. Schrettl, M. *et al.* Distinct Roles for Intra- and Extracellular Siderophores during *Aspergillus fumigatus* Infection. *PLoS Pathog* **3**, e128 (2007).
5. Gressler, M. *et al.* Phytotoxin production in *Aspergillus terreus* is regulated by independent environmental signals. *eLife* **4**, e07861 (2015).
6. Misslinger, M., Hortschansky, P., Brakhage, A. A. & Haas, H. Fungal iron homeostasis with a focus on *Aspergillus fumigatus*. *Biochimica et Biophysica Acta (BBA) - Molecular Cell Research* **1868**, 118885 (2021).
7. Yasmin, S. *et al.* Mevalonate governs interdependency of ergosterol and siderophore biosyntheses in the fungal pathogen *Aspergillus fumigatus*. *Proc Natl Acad Sci U S A* **109**, E497–E504 (2012).
8. Happacher, I., Aguiar, M., Yap, A., Decristoforo, C. & Haas, H. Fungal siderophore metabolism with a focus on *Aspergillus fumigatus*: impact on biotic interactions and potential translational applications. *Essays in Biochemistry* **67**, 829–842 (2023).
9. Blatzer, M. *et al.* SidL, an *Aspergillus fumigatus* Transacetylase Involved in Biosynthesis of the Siderophores Ferricrocin and Hydroxyferricrocin ▿. *Appl Environ Microbiol* **77**, 4959–4966 (2011).
10. Caballero, P. *et al.* *Aspergillus fumigatus* SidF is a dual substrate acyltransferase involved in biosynthesis of both fusarinine- and ferrichrome-type siderophores. 2024.08.20.608788 Preprint at https://doi.org/10.1101/2024.08.20.608788 (2024).
11. Poonsiri, T. *et al.* SidF, a dual substrate N5-acetyl-N5-hydroxy-L-ornithine transacetylase involved in *Aspergillus fumigatus* siderophore biosynthesis. *Journal of Structural Biology: X* **11**, 100119 (2025).
12. Gründlinger, M. *et al.* Fungal siderophore biosynthesis is partially localized in peroxisomes. *Mol Microbiol* **88**, 862–875 (2013).
