# Supplementary material for: Siderophore production by the lichen fungus Xanthoria parietina supports its algal symbiont
Source: Nat Commun. 2026 Jul 2;17:8234. doi: 10.1038/s41467-026-74988-9 (PMC13462969; doi:10.1038/s41467-026-74988-9)
Supplement: Supplementary file 2 [file 41467_2026_74988_MOESM2_ESM.pdf]

Description of Additional Supplementary Files:

Supplementary Data 1: Iron homeostatic mechanisms are highly conserved across lichen mycobiont species.
