# Supplementary material for: Siderophore production by the lichen fungus Xanthoria parietina supports its algal symbiont
Source: Nat Commun. 2026 Jul 2;17:8234. doi: 10.1038/s41467-026-74988-9 (PMC13462969; doi:10.1038/s41467-026-74988-9)
Supplement: Supplementary file 4 [file 41467_2026_74988_MOESM5_ESM.pdf]

nature portfolio

Peer Review File

Siderophore production by the lichen fungus *Xanthoria parietina* supports its algal symbiont

Corresponding Author: Professor Hubertus Haas

This file contains all reviewer reports in order by version, followed by all author rebuttals in order by version.

Version 1:

Reviewer comments:

Reviewer #1

(Remarks to the Author)
The authors present a manuscript in which they reveal the genetic repertoire for siderophore production in the lichen mycobiont Xanthoria parietina, including factors for reductive iron assimilation, detoxification, and regulation. They show that ferrichrome is formed as a hydroxamate-type siderophore via a non-ribosomal biosynthetic pathway. The involvement of the XpSidC NRPS in the synthesis of Xanthoria ferrichrome was also confirmed via heterologous expression in Aspergillus. These findings are fundamentally similar to the repertoire of the model organism Aspergillus fumigatus, but are analyzed here for the first time in the lichen context and also reveal some special features, e.g. with regard to the domain architecture and the siderophore subtype. The authors also demonstrate substrate-dependent regulation of the genes and prove their role in mutualistic nutrient exchange and growth promotion of the phycobiont Trebouxia. The data are easy to understand and the conclusions are generally justified. The study will be of interest to the specific scientific community interested in lichen or siderophores, although the findings are not particularly surprising from a biosynthetic or regulatory perspective.

Specific comments:

1. Fig. 1a I was missing a panel with A.fumigatus wild type growth for comparison. Fig. 1b summarizes published data from the literature partially overlapping with actual data in Fig. 1a. I would omit this part of the figure, it is rather confusing and redundant.

2. Fig. 1-2 The authors show a more pronounced siderophore mutant complementation for the lichen thallus compared to the axenic mycobiont as well as higher amounts of ferrichrome produced by the thallus as shown in Figure 2. This can have multiple reasons, e.g. the presence of further ferrichrome producers in the thallus (as suggested by the authors), a transcriptional upregulation in the lichen thallus, or presence of further siderophores (for the bioassays). It would be valuable for the study to dissect the different possibilities, e.g. compare transcription of siderophore genes in the axenic versus the lichen context. Another possibility would be to use MS Imaging, which however is not always available (could also clarify whether there is siderophore-complexed iron uptake). As the functional analysis of siderophore context is central for this study, a simple comparison of bioassays seems somewhat incomplete.

3. Fig. 6 The acquisition of the samples and RNAseq data are not a genuine part of the reported work, but was done previously. It is good that the authors are taking the advantage of the already generated data and gain additional insight from the already published dataset. As mentioned above a comparison of the axenic mycobiont and the lichen thallus would have been more valuable for this study.

4. It is not clear if the particular strain of T. decolorans and X. parietina comes from the same sample. For microbial interactions, species level is not detailed enough, as members of the same species could easily interact differently with the mycobiont or vice versa.

5. The authors should include bioinformatic information on potential iron transport systems in lichen phycobionts as shown in Supplementary S3 in their results section and not only their discussion. They should also consider testing the presence of these genes in the particle T. decolorans isolate used for the supplementation assays in Figure 7. These results are important for the interpretation of the supplementation assay.

6. 417ff. The discussion on mineral weathering is a bit out of scope. No original experiments are provided and the statement is very superficial.

Reviewer #2

(Remarks to the Author)
This manuscript addresses an understudied aspect of lichen biology with potential implications for understanding nutritional exchanges in symbiotic systems. Specifically, the study presents a novel investigation into siderophore-mediated iron acquisition in the lichen symbiosis between Xanthoria parietina (mycobiont) and its photosynthetic partner Trebouxia sp. (photobiont). The authors demonstrated that ferrichrome, a siderophore produced by the fungal partner, facilitates iron acquisition by the freely living (i.e. not symbiotic) algal cells and their growth. Given the extremely slow growth rates of both partners and the technical challenges inherent to studying lichen biology, this work represents a valuable contribution as the first detailed examination of siderophore function in a lichen system. However, several substantial concerns need to be addressed before the manuscript can be recommended for publication in Nature Communications.

Major Issues
1. The characterization of the NRPS (non-ribosomal peptide synthetase) enzymes requires substantial strengthening. Given the conserved domain architecture of NRPS enzymes, the authors should construct comprehensive phylogenetic trees to examine the homologous relationships by assessing the monophyly of the examined NRPS genes and comparing these phylogenies with species trees to evaluate congruence.
In addition, it would be more informative to provide a broader comparative genomic analysis of SIA and RIA genetic repertoires across diverse lichen-forming fungi, encompassing various growth forms (e.g., crustose lichens vs. macrolichens). This may contribute to understanding lichen evolutionary biology and strengthening claims about the functional significance of these pathways.

2. It seems reasonable to assume the concrete conditions may be iron-poor conditions, compared to organic substrates. However, it is not sure they really are. Both conditions may be lacking iron. Some supporting data may be required (iron contents or gene expression levels of iron-responsive genes, if any). Is iron content in concrete versus bark substrates available. If not, expression levels of established iron-responsive marker genes should be quantified to confirm iron limitation. While statistical significance is reported for siderophore-related genes between substrate conditions, the actual differences in TPM values appear modest. Iron-responsive genes, such as, SreA, HapX showed no differential expression between conditions.

3. The key conclusion—that fungal ferrichrome is delivered to and utilized by the photobiont in the intact lichen—is primarily based on in vitro observations. While these experiments demonstrate that exogenous ferrichrome can enhance algal growth under laboratory conditions, this does not necessarily reflect the physiological reality within the symbiotic thallus. To strengthen this central claim, the authors should provide direct evidence of ferrichrome localization within algal cells in intact lichen thalli. 3D-MALDI imaging mass spectrometry, while technically challenging, would be highly informative. If not feasible to perform this analysis, the author need to discuss alternative interpretations and the limitations of extrapolating from in vitro to in vivo conditions more explicitly.

Minor Issues
1. Several paragraphs in the Result sections contain background information more appropriate for the Introduction. Lines 245-264 and Lines 304-310. Please restructure these sections to maintain focus on the experimental findings.

2. The legend for Supplementary Table S3 appears unclear or potentially incorrect. Please verify and revise for accuracy.

Reviewer #3

(Remarks to the Author)
In their article entitled « Iron for two: siderophore production by the lichen fungus Xanthoria parietina supports it algal symbiont", Happacher and collaborators elucidate one mechanism by which the lichen-forming fungus X. Parietina acquire iron for its metabolism and for its algal symbiont Trebouxia decolorans. The article relies on series of robust and complementary experimentations which provide a convincing demonstration of the role of fungal ferrichrome for both mycobiont and algae development. The biosynthesis pathway uncovered in the mycobiont is not novel, as it is largely shared among lichenous and non lichenous fungi, but the description of its involvement in lichen biology is a valuable contribution to the field. The manuscripts reads well and is well illustrated. A few points would deserve to be discussed. First of all, one would ask what is the relative contribution of the remaining members of the lichen microbiome for iron acquisition; as the authors showed in a previous article (Tagirdzhanova et al. 2025), and also shown by other groups, lichens also harbor a complex microbial community, including bacteria. A large diversity of siderophores is produced by bacteria and

including siderophores with higher affinities than ferrichrome, and one may wonder whether the mechanism uncovered here acts alone, or is one among many others siderophores produced together by the microbiota. Such question is never mentioned in the manuscript and the way it is written could drive the false impression for non specialist readers that algal iron acquisition solely rely on the fungal partner, which still needs to be demonstrated. A second point which would deserve additional discussion are the transcriptomic results. As noticed by the authors, but not discussed, one may wonder why SidL homolog is downregulated on concrete while all other genes are up. It is also surprising to see that the level of expression of SidC is very low compared to other genes; these points should be mentioned and discussed. Incorporating the level of expression and fold changes between concrete vs bark into the figure 4 may improve the interpretability of the transcriptomic results. In addition, information about the meaning of sampling codes for transcriptomic data should be provided in the legend of Supplemental Table S1 (i.e what's the difference between XB and XT and so one, without having to go to the original article). Lastly, it is a bit surprising to find results presented in the discussion only (Supplemental Table S3): a result section focusing on the search of homologs of the pathway in others organisms would make sense. It should be noted that the percentage of identity for some species are rather low and the interpretation may require caution.

Minor points:
Line 114-115: part of the sentence is missing
Line 119 "In summary …" I do not see in the previous sentences any indication that siderophore are involved in mutualistic interactions; the fact tha symbionts produce siderophore for their iron supply does not mean that it is involved in the symbiosis.
Table 1. Is the % cov for SitT is really of 131.2 % ? or is it a typo .
Suppl Table 3. A word is missing in the title of the table: "Homologous proteins involved or potentially involved in the microalgae Trebouxia sp., Chlamydomonas reinhardtii, Dunaliella and Phaeodactylum tricornutum »

Reviewer #4

(Remarks to the Author)
In Happacher et al., the authors explore, for the first time, the iron scavenging mechanisms of a lichen, Xanthoria parietina. The authors use a powerful combination of approaches, ranging from bioassays with Aspergillus fumigatus knockout mutants and X. parietina extracts, a BLAST-based genomic investigation of proteins in the putative X. parietina iron uptake and detoxification regulatory mechanisms, CESI-MS/MS identification of the putative ferrichrome produced by the X. parietina mycobiont, and finally some evidence of the mycobiont-derived ferrichrome's function in the X. parietina photobiont, Trebouxia decolorans.

Overall, the hypotheses are highly novel and well-investigated, and the work therefore warrants publication. The work is well-written and largely well-ordered and clear. The methodologies used are sound and state of the art, and very much cutting-edge when it comes to lichenological work. The methods section appear sufficiently detailed.

I have some minor suggestions and comments that I will outline below. My primary points of criticism/request for additional explanations or alternate interpretations have to do with the algal growth assay in Fig. 7 and its conclusions. Although the use of the mycobiont-derived siderophore by the alga to some degree appears evident, I am unconvinced so far by the specific interpretations made by the authors of the experimental result, and of the soundness of these specific data for Fig. 7 (i.e. the health of the algal cultures). However, this may be a difference in overall health of axenic cultures of T. decolorans relative to other Trebouxia species; if so, perhaps the authors can refer to additional literature demonstrating that these values are not abnormal for T. decolorans.

L 99: "In RIA, ferric iron..." This should be the beginning of a new paragraph.

LL 114-115: "Root tissues…" This sentence is missing a word (perhaps "contain").

LL 132-133: "Compared to…" Shouldn't we expect that X. parietina, which often grows on relatively more heavy metal-polluted environments, produces less variable or makes less use of siderophores or generally engages in less iron scavenging than other lichen species that grow in iron-poor environments (e.g. many arctic or antarctic species)?

If this logic is deficient in the authors' eyes, perhaps they could elaborate more on the rationale for using X. parietina for this initial study. Below the argument seems to be made that human-made habitats like concrete are iron poor, and that X. parietina's ready colonization of these habitats implies a robust siderophore production for iron scavenging, but this should be stated clearly here.

L 147: "…and potential for RIA and iron detoxification." Mention of this could be made above in the rationale for using X. parietina. Additionally, mention of iron detoxification and its relationship to sideorphore production could be made more clearly in the earlier paragraphs.

L 153: Maybe a line should be added here to describe explicitly what siderophores Sit2 is the expected transporter of, relative to Sit1 (my understanding was that both have overlapping transport ability and some specific transport ability).

L 165/Fig. 1: "negativ control" -> negative control. I would also recommend the checks and Xs in panel B be made in different colors.

L 240/Fig. 4: The algal side of this schematic seems misleadingly incomplete, even in its largely hypothetical state. Is there a reason the authors do not assume/do not explore the possibility that iron is ferried by larger transport forms after processing in the mycobiont cells to the photobiont?

L 297/Fig. 6: Maybe the authors should show some alternative genes/pathways not expected to be upregulated in concrete vs bark? What if this is just generally more biological activity in concrete relative to bark, sampling time, time of year, etc.?

L 330/Fig. 7: Even though Fv/Fm values for lichen-forming algae are generally lower than normal for plants, these values all seem particularly very low. Even the cells receiving the ferrichrome-iron chelate are barely above 0.3, half of the values we usually see in lichen-forming Trebouxia cultures in the lab. Are the authors really sure that these cells were comparable/healthy enough to draw reasonable conclusions from? Or are these values to be expected from T. decolorans?

In addition, there is no statistical test seemingly being applied here. It is hard to tell if there is any meaningful biological difference between the iron-free ferrichrome and the bound version of the ferrichrome, perhaps, again, because the cells are so sickly/unhealthy to begin with, or perhaps because the iron-scavenging phenotype is not as clearly beneficial as the authors are claiming?

LL 388-389: "…suggesting that ferrichrome-iron chelates are most likely utilized by internalization." I am not clear on the authors' argument for why the iron-free version of the ferrichrome causing essentially the same growth potential in Trebouxia as the ferrichrome-iron chelate is consistent with the idea that the Trebouxia is using the mycobiont ferrichrome for iron scavenging. If anything, this seems to imply that the ferrichrome is somehow required by the photobiont for growth (as they mention in the subsequent sentences), but that iron scavenging itself is not generally required for growth of the photobiont.

Otherwise, presumably the iron-free version of the ferrichrome would be expected to cause severe growth defects relative to the ferrichrome-iron chelate. If the authors disagree with this logic, I would be happy to read their argument here more clearly.

Best,
Henrique Valim
Senckenberg BiK-F
Frankfurt am Main, Germany

Reviewer #5

(Remarks to the Author)
I co-reviewed this manuscript with one of the reviewers who provided the listed reports. This is part of the Nature Communications initiative to facilitate training in peer review and to provide appropriate recognition for Early Career Researchers who co-review manuscripts.

Version 2:

Reviewer comments:

Reviewer #2

(Remarks to the Author)
The authors have addressed most of the concerns raised during the review process. I believe the revisions have significantly strengthened the manuscript, and the data now better support the conclusions. I am pleased to recommend this article for publication after some minor issues.

Minor issues: The author added a phylogenetic tree of SidC (Fig. 3c). The figure could be improved by removing bootstrap values smaller than certain values, leaving highly supported ones. The font for BS values can be bigger such that it may be more readable. Is the tree mid-point rooting or outgroup-rooting. The ancestral branch looks incomplete.

Open Access This Peer Review File is licensed under a Creative Commons Attribution 4.0 International License, which permits use, sharing, adaptation, distribution and reproduction in any medium or format, as long as you give appropriate credit to the original author(s) and the source, provide a link to the Creative Commons license, and indicate if changes were made.
In cases where reviewers are anonymous, credit should be given to 'Anonymous Referee' and the source.
The images or other third party material in this Peer Review File are included in the article's Creative Commons license, unless indicated otherwise in a credit line to the material. If material is not included in the article's Creative Commons license and your intended use is not permitted by statutory regulation or exceeds the permitted use, you will need to obtain permission directly from the copyright holder.
To view a copy of this license, visit https://creativecommons.org/licenses/by/4.0/

Point-by-point response (in blue)

Reviewer #1 (Remarks to the Author):

The authors present a manuscript in which they reveal the genetic repertoire for siderophore production in the lichen mycobiont Xanthoria parietina, including factors for reductive iron assimilation, detoxification, and regulation. They show that ferrichrome is formed as a hydroxamate-type siderophore via a non-ribosomal biosynthetic pathway. The involvement of the XpSidC NRPS in the synthesis of Xanthoria ferrichrome was also confirmed via heterologous expression in Aspergillus. These findings are fundamentally similar to the repertoire of the model organism Aspergillus fumigatus, but are analyzed here for the first time in the lichen context and also reveal some special features, e.g. with regard to the domain architecture and the siderophore subtype. The authors also demonstrate substrate-dependent regulation of the genes and prove their role in mutualistic nutrient exchange and growth promotion of the phycobiont Trebouxia. The data are easy to understand and the conclusions are generally justified. The study will be of interest to the specific scientific community interested in lichen or siderophores, although the findings are not particularly surprising from a biosynthetic or regulatory perspective.

We thank the reviewer for the positive feedback and for the valuable suggestions to improve our manuscript. While nutrient exchange between the mycobiont and photobiont is often assumed in lichen symbioses, experimental evidence for the physiological availability of specific micronutrients has remained scarce, particularly for iron.

Iron is a pivotal element for photosynthesis, respiration, and redox metabolism, yet its functional accessibility to the photobiont has not been demonstrated in any lichen system previously. In this study, we identify ferrichrome as the siderophore produced by the lichen mycobiont and show that it is released into the extracellular environment. We further demonstrate that ferrichrome is sufficient to sustain photobiont growth when provided as the sole iron source, establishing its physiological relevance beyond pathway annotation or genomic inference. When considered together, these results provide functional support for siderophore-mediated iron provisioning as a biologically meaningful interface in the lichen symbiosis, extending concepts derived from free-living fungi into a mutualistic context. We feel this is highly significant, given the paucity of physiological evidence for nutrient exchange in lichens.

Specific comments:

1. Fig. 1a I was missing a panel with A.fumigatus wild type growth for comparison.

Wild type growth for *A. fumigatus* is now shown for comparison and the text has been adapted accordingly.

Fig. 1b summarizes published data from the literature partially overlapping with actual data in Fig. 1a. I would omit this part of the figure, it is rather confusing and redundant.

We are grateful for this comment and Fig. 1b has been removed.

2. Fig. 1-2 The authors show a more pronounced siderophore mutant complementation for the lichen thallus compared to the axenic mycobiont as well as higher amounts of ferrichrome produced by the thallus as shown in Figure 2. This can have multiple reasons, e.g. the presence of further ferrichrome producers in the thallus (as suggested by the authors), a transcriptional upregulation in the lichen thallus, or presence of further siderophores (for the bioassays). It would be valuable for the study to dissect the different possibilities, e.g. compare transcription of siderophore genes in the axenic versus the lichen context. Another possibility would be to use MS Imaging, which however is not always available (could also clarify whether there is siderophore-complexed iron uptake). As the functional analysis of siderophore context is central for this study, a simple comparison of bioassays seems somewhat incomplete.

Indeed, the ferrichrome content was higher in the lichen thallus than in the axenic mycobiont culture, as indicated by the bioassay as well as by MS and MS/MS analyses. The quantitative difference in the MS analyses was not explicitly stated in the original version of the manuscript but has now been included. As noted by the reviewer, this difference could arise from multiple factors, for example the presence of additional ferrichrome-producing organisms in the lichen thallus or an upregulation of ferrichrome biosynthesis in the symbiotic state. Although MS imaging would be an interesting approach to localize metabolites in situ, it cannot discriminate between these two possibilities. Moreover, we unfortunately do not have direct access to MS imaging and utilisng such an approach would require a new long-term collaborative project to be established which would be beyond the scope of a manuscript revision.

From our perspective, the absolute level of ferrichrome production in axenic culture should not be overinterpreted, because axenic culturing represents a highly artificial condition. The transition to the symbiotic state is accompanied by shifts in metabolite accumulation between free-living fungi in culture and their lichenized counterparts, as exemplified for photosynthetic pigments and antioxidants (see PMID: 15710882). Nevertheless, inclusion of the axenic culture was important, as it unequivocally demonstrates that the mycobiont itself is capable of producing ferrichrome in the absence of other organisms, which cannot be resolved in the intact thallus. In summary, in our system the axenic culture is primarily informative for qualitative rather than quantitative aspects of metabolite production. We have, however, now clarified this aspect in the Discussion section of the manuscript in response to the thoughtful comment of the reviewer (lines 469-478).

3. Fig. 6 The acquisition of the samples and RNAseq data are not a genuine part of the reported work, but was done previously. It is good that the authors are taking the advantage of the already generated data and gain additional insight from the already published dataset. As mentioned above a comparison of the axenic mycobiont and the lichen thallus would have been more valuable for this study.

We agree that such a comparison would be informative in a broader systems-level framework. However, this was not the central aim of the present study, which focuses on demonstrating that the fungal partner is capable of autonomous siderophore production and that this compound is available in the symbiotic environment. A direct transcriptomic comparison would additionally require matched axenic and symbiotic datasets generated under strictly comparable conditions, which are currently not available and would constitute a substantial independent study. We have revised the Discussion to acknowledge this limitation in our study and to outline how future transcriptomic comparisons could further refine the relative contributions of the mycobiont to iron acquisition (lines 478-480).

4. It is not clear if the particular strain of T. decolorans and X. parietina comes from the same sample. For microbial interactions, species level is not detailed enough, as members of the same species could easily interact differently with the mycobiont or vice versa.

We thank the reviewer for this comment and would like to clarify an implicit assumption underlying this concern. *Trebouxia decolorans* and *Xanthoria parietina* were isolated from an intact lichen, although not from the same lichen thallus used for some of the experiments reported. As noted in the Methods, the axenic photobiont and mycobiont cultures used in this study originate from established culture collections. Obtaining paired, axenic symbiont cultures from a single lichen thallus is an exceptionally time-consuming process that typically requires several years to achieve and is beyond the scope of this study. We have taken advantage of some of the only available examples of purified mycobiont and photobionts and associated genomic resources for this study. Our experimental design deliberately contrasts axenic, free-living cultures with naturally lichenized thalli collected from the field, with the aim of identifying metabolic shifts associated with the symbiotic state per se, rather than strain-specific interaction effects. This approach is widely used in lichen physiology and metabolomics and we believe is appropriate for addressing the questions posed here. We agree that intra-specific variation among strains can influence interaction outcomes. However, resolving strain-level specificity was not an objective of this study and would

require a fundamentally different experimental framework. We have now clarified this in the Methods to make this point explicitly, in view of the reviewer concern (lines 595-596).

5. The authors should include bioinformatic information on potential iron transport systems in lichen phycobionts as shown in Supplementary S3 in their results section and not only their discussion. They should also consider testing the presence of these genes in the particle T. decolorans isolate used for the supplementation assays in Figure 7. These results are important for the interpretation of the supplementation assay.

The *Trebouxia* sp. data originally presented in Supplementary Table S3 have now been incorporated into the Results section as Table 2 in the main text. We consider that the bioinformatic analysis of the other microalgae species does not integrate well in the main manuscript and therefore remains in the supplementary material. To streamline the presentation, only the homolog with the highest significance (lowest E-value) is shown for each isolate, while the complete dataset is still provided in Supplementary Table S4. Unfortunately, the genome sequence of the used isolate is not available, which limits the analysis of siderophore- and iron-uptake genes that we are able to undertake.

6. 417ff. The discussion on mineral weathering is a bit out of scope. No original experiments are provided and the statement is very superficial.

The discussion on the potential role of siderophores in mineral weathering has been removed to provide greater focus to the discussion section, as suggested.

Reviewer #2 (Remarks to the Author):

This manuscript addresses an understudied aspect of lichen biology with potential implications for understanding nutritional exchanges in symbiotic systems. Specifically, the study presents a novel investigation into siderophore-mediated iron acquisition in the lichen symbiosis between Xanthoria parietina (mycobiont) and its photosynthetic partner Trebouxia sp. (photobiont). The authors demonstrated that ferrichrome, a siderophore produced by the fungal partner, facilitates iron acquisition by the freely living (i.e. not symbiotic) algal cells and their growth. Given the extremely slow growth rates of both partners and the technical challenges inherent to studying lichen biology, this work represents a valuable contribution as the first detailed examination of siderophore function in a lichen system. However, several substantial concerns need to be addressed before the manuscript can be recommended for publication in Nature Communications.

We are very grateful to the reviewer for recognizing the novelty of this study and the significant technical challenges inherent in studying lichen biology. The thoughtful and constructive feedback has greatly contributed to improving the manuscript.

Major Issues
1. The characterization of the NRPS (non-ribosomal peptide synthetase) enzymes requires substantial strengthening. Given the conserved domain architecture of NRPS enzymes, the authors should construct comprehensive phylogenetic trees to examine the homologous relationships by assessing the monophyly of the examined NRPS genes and comparing these phylogenies with species trees to evaluate congruence. In addition, it would be more informative to provide a broader comparative genomic analysis of SIA and RIA genetic repertoires across diverse lichen-forming fungi, encompassing various growth forms (e.g., crustose lichens vs. macrolichens). This may contribute to understanding lichen evolutionary biology and strengthening claims about the functional significance of these pathways.

We have now conducted a comprehensive *in silico* analysis of potential iron-homeostatic proteins in an additional 53 lichen mycobionts species for which genome sequences and annotated proteomes are available, representing 43 genera within the Pezizomycota (new Supplementary Table S3). The dataset was dominated by members of the Lecanoromycetes/OSLEUM clade (49 species), with additional representation from two Dothideomycetes species (*Trypethelium eluteriae, Viridothelium virens*), one Eurotiomycetes species (*Endocarpon pusillum*), and one Leotiomycetes species (*Caeruleum heppii*). The 49 OSLEUM clade species spanned three subclasses - Lecanoromycetidae (34 species), Ostropomycetidae (13 species), and Umbilicariomycetidae (2 species) - and comprised 12 orders and 19 families. Among these taxa, 26 form macrolichens like *X. parietina*, whereas 25 are crustose lichens.

Among the 53 mycobiont species analyzed, 41 contained a SidC homolog, 41 contain a SidA homolog, and 51 a SidL homologue, with all species possessing at least one of these genes. Given potential incompleteness in gene and protein annotations, it is likely that all species are capable of siderophore biosynthesis. No homologues of *SidD* were detected in any species. The presence of SidC and/or SidL homologues indicates that most, if not all, analyzed mycobionts exclusively produce ferrichrome-type siderophores. In 38 of the 39 species harboring both SidA and SidC homologues, the encoding genes are genomically clustered, as observed in *X. parietina*. 50 species possessed at least on putative siderophore transporter. All 53 species possessed a FetC homolog, and 50 also contained an FtrA homolog, with the corresponding genes genomically clustered, mirroring the organization observed in *X. parietina*. Homologues of FetB and HapX were detected in all species, CccA homologues in 49 species, and SreA homologues in 47 species. Taken together, these findings indicate that the iron homeostatic system is highly conserved across the lichen mycobionts analyzed. There was no obvious difference between macrolichens and crustose lichens.

Phylogenetic analysis of the identified 41 mycobiont SidC homologues together with 37 homologues from non-lichenized fungal species known to produce ferrichrome-type siderophores showed that 33 of the 37 OSLEUM-clade sequences form a distinct subclade (new Fig. 3c). The remaining four OSLEUM-clade homologues, all from Peltigerales species, as well as homologues from non-OSLEUM mycobionts (Dothideomycetes), grouped with sequences from non-lichenized fungi (Fig. 3c). Consistent with this phylogenetic pattern, the 33 OSLEUM-clade homologues in the subclade are share the compact NRPS domain architecture characteristic of *X. parietina*, whereas the remaining mycobiont homologues exhibit an architecture more similar to that of *A. fumigatus* (new Supplementary Fig. S3). These results support a monophyletic origin of compact-architecture *SidC* homologues within the OSLEUM clade, with the Peltigerales homologues constituting a notable exception.

These data are given in a new addition to the Results section. Due to inclusion of these new data, the original Supplementary Table S2 has been removed.

2. It seems reasonable to assume the concrete conditions may be iron-poor conditions, compared to organic substrates. However, it is not sure they really are. Both conditions may be lacking iron. Some supporting data may be required (iron contents or gene expression levels of iron-responsive genes, if any). Is iron content in concrete versus bark substrates available. If not, expression levels of established iron-responsive marker genes should be quantified to confirm iron limitation. While statistical significance is reported for siderophore-related genes between substrate conditions, the actual differences in TPM values appear modest. Iron-responsive genes, such as, SreA, HapX showed no differential expression between conditions.

We agree with the reviewer that iron availability, rather than total iron content, is the biologically relevant parameter. While direct measurements of bioavailable iron in bark versus concrete substrates are currently unavailable, it is biologically plausible that organic substrates provide higher iron bioavailability due to the presence of iron-containing biomolecules and ongoing metabolic turnover, whereas mineral substrates largely lack such pools.

The modest differences in TPM values observed for siderophore-related genes suggest that any differences in iron availability between substrates are likewise modest. This interpretation is

consistent with the generally low growth rates of lichens, which are expected to attenuate transcriptional amplitudes even under changing environmental conditions. Importantly, in filamentous fungi such as *Aspergillus fumigatus*, only a very limited set of genes responds almost exclusively to iron availability in a manner inverse to iron acquisition genes such as *cccA*, which encodes for a vacuolar transporter mediating iron storage and iron detoxification. Other iron-associated metabolic genes (e.g. *hemA*, *cycA*, *acoA*, *nbp35*) are subject to additional regulatory inputs and therefore the interpretation of their expression is difficult. The lack of differential expression of *cccA* (Supplementary Table S1), together with the absence of a response in the key iron regulator genes *sreA* and *hapX* (Supplementary Table S1), suggests that both substrates likely represent similarly iron-limited environments, with only subtle differences in bioavailability. Nevertheless, the expression data indicate site-dependent regulation of the siderophore biosynthesis. We have therefore included the expression of *cccA, sreA* and *hapX* in the Results section.

To further investigate potential substrate-dependent ferrichrome production by the *X. parientina* mycobiont, we analyzed ferichrome contents in three samples growing on bark and three on granite (new Fig. 6b). Ferrichrome content varied 4.7-fold, ranging from 1.8 pmol (1.3 ng) mg^{-1} dry mass in a bark sample to 8.5 pmol (6.3 ng) mg^{-1} dry mass in a granite sample. Although the mean ferrichrome content in granite samples was 1.8-fold higher than in bark samples, the difference was not statistically significant. Nevertheless, these results provide evidence that ferrichrome levels of *X. parietina* lichen thalli vary across substrates or sites, most likely reflecting differences in iron bioavailability. These results have been included in the main text. The Discussion has been revised to frame siderophore-related transcriptional changes and siderophore production as indicative of substrate-associated physiological adjustment rather than as direct evidence for organic versus organic substrates.

3. The key conclusion—that fungal ferrichrome is delivered to and utilized by the photobiont in the intact lichen—is primarily based on in vitro observations. While these experiments demonstrate that exogenous ferrichrome can enhance algal growth under laboratory conditions, this does not necessarily reflect the physiological reality within the symbiotic thallus. To strengthen this central claim, the authors should provide direct evidence of ferrichrome localization within algal cells in intact lichen thalli. 3D-MALDI imaging mass spectrometry, while technically challenging, would be highly informative. If not feasible to perform this analysis, the author need to discuss alternative interpretations and the limitations of extrapolating from in vitro to in vivo conditions more explicitly.

We thank the reviewer for raising this important point. While direct spatial visualization of ferrichrome within algal cells of intact lichen thalli would certainly be informative, our conclusions do not depend on such localization. Instead, they rest on a functional and mechanistic demonstration that ferrichrome alone is sufficient to promote photobiont growth in the complete absence of extracellular iron reduction. As shown in Fig. 7, algal growth is abolished without iron, whereas abundant growth is restored when ferrichrome is provided as the sole iron source. This unambiguously excludes indirect, reductive iron acquisition pathways and necessarily implies direct uptake and intracellular utilization of ferrichrome-derived iron by the photobiont. Crucially, this establishes physiological relevance through causality rather than spatial correlation, thereby extending beyond descriptive or *in vitro* associations. Additional localization experiments, while valuable for future studies, would therefore refine rather than alter the central conclusion of this work, which is already firmly supported by functional evidence. We would like to carry out analytical biochemical analysis on lichen thalli, but we are also aware that 3D-MALDI imaging mass spectrometry would not allow to discriminate extra- and intracellular localization of ferrichrome because cells have to be destroyed to make ferrichrome visible to mass spectrometry. So, although we recognize the reviewers' concern, the approach may not be as informative as suggested due to this limitation. We have made a revision to the text to highlight the limitations in our study and the need for direct analysis of the lichen thallus (lines 562-565).

Minor Issues
1. Several paragraphs in the Result sections contain background information more appropriate for the Introduction. Lines 245-264 and Lines 304-310. Please restructure these sections to maintain focus on the experimental findings.

The indicated paragraphs in the Results section (lines 245–264 and 304–310) have been restructured to remove background information and to maintain a clearer focus on the experimental findings.

2. The legend for Supplementary Table S3 appears unclear or potentially incorrect. Please verify and revise for accuracy.

The legend for Supplementary Table S3(now Table S4) has been carefully checked and revised for clarity and accuracy.

Reviewer #3 (Remarks to the Author):

In their article entitled « Iron for two: siderophore production by the lichen fungus Xanthoria parietina supports it algal symbiont", Happacher and collaborators elucidate one mechanism by which the lichen-forming fungus X. Parietina acquire iron for its metabolism and for its algal symbiont Trebouxia decolorans. The article relies on series of robust and complementary experimentations which provide a convincing demonstration of the role of fungal ferrichrome for both mycobiont and algae development. The biosynthesis pathway uncovered in the mycobiont is not novel, as it is largely shared among lichenous and non lichenous fungi, but the description of its involvement in lichen biology is a valuable contribution to the field. The manuscripts reads well and is well illustrated. A few points would deserve to be discussed.

We are very grateful to the reviewer for their positive assessment and for the thoughtful and constructive feedback, which have greatly contributed to improving the manuscript.

First of all, one would ask what is the relative contribution of the remaining members of the lichen microbiome for iron acquisition; as the authors showed in a previous article (Tagirdzhanova et al. 2025), and also shown by other groups, lichens also harbor a complex microbial community, including bacteria. A large diversity of siderophores is produced by bacteria and including siderophores with higher affinities than ferrichrome, and one may wonder whether the mechanism uncovered here acts alone, or is one among many others siderophores produced together by the microbiota. Such question is never mentioned in the manuscript and the way it is written could drive the false impression for non specialist readers that algal iron acquisition solely rely on the fungal partner, which still needs to be demonstrated.

We agree that lichens harbour complex microbial communities and that bacteria associated with the lichen microbiome will be capable of producing a diverse array of siderophores, including compounds with high iron-binding affinities. Our study does not aim to quantify the relative contributions of all potential iron-acquisition pathways operating within the intact lichen holobiont. Importantly, however, we clearly demonstrate that ferrichrome is produced by an axenically cultured mycobiont, released into the extracellular environment, and directly utilizable by the photobiont. This establishes a fungal-derived, microbiome-independent mechanism for iron provisioning between the two principal symbiotic partners and demonstrates that bacterial partners are not required for this process to occur. We do not suggest that this mechanism acts in isolation in natural thalli, nor that algal iron acquisition relies exclusively on the fungal partner. Rather, our data identify a specific, previously unresolved fungal–algal iron transfer route that likely operates alongside additional microbiome-mediated processes. The focus of the present study is explicitly on the mycobiont–photobiont interaction, and a comprehensive analysis of iron acquisition across the entire lichen microbiota – which may contain about 1000 different organisms – is beyond the scope of this work. However, we have amended the text to clarify this aspect of the study and its limitations (lines 565-567).

A second point which would deserve additional discussion are the transcriptomic results. As noticed by the authors, but not discussed, one may wonder why SidL homolog is downregulated on concrete while all other genes are up.

Indeed, the opposite regulation of the gene encoding SidL compared to the other SIA genes is surprising. Possibly, the SidL-encoding gene is subject to additional regulatory circuits in addition to iron. We have included a discussion of this point in the text (lines 511-514).

It is also surprising to see that the level of expression of SidC is very low compared to other genes; these points should be mentioned and discussed. Incorporating the level of expression and fold changes between concrete vs bark into the figure 4 may improve the interpretability of the transcriptomic results.

As suggested, we have incorporated expression levels normalized to mean transcripts per million (TPM) across all samples into the heatmap in Fig. 6. The seemingly low expression of *SidC* may be attributable to its unusually large transcript (>10,000 nucleotides) relative to other genes. In addition, the encoded protein may exhibit high stability. Notwithstanding, the protein is functionally active, as indicated by detection of the metabolite produced by the enzyme."

In addition, information about the meaning of sampling codes for transcriptomic data should be provided in the legend of Supplementary Table S1 (i.e what's the difference between XB and XT and so one, without having to go to the original article).

A detailed description of the samples has now been provided in legend of Supplementary Table S1.

Lastly, it is a bit surprising to find results presented in the discussion only (Supplemental Table S3): a result section focusing on the search of homologs of the pathway in others organisms would make sense. It should be noted that the percentage of identity for some species are rather low and the interpretation may require caution.

The *Trebouxia* sp. data originally presented in Supplementary Table S3 have been incorporated into the Results section as Table 2 in the main text. We consider that the bioinformatic analysis of the other microalgae species does not integrate well here and therefore remains in the supplementary material. To ensure presentation is succinct, only the homolog with the highest significance (lowest E-value) is shown for each isolate, while the complete dataset is still provided in Supplementary Table S3 (now Table S4). The relatively low percentage identity observed for some homologues is likely due to the use of proteins from phylogenetically distant organisms as bait (e.g., fungal RIA proteins versus *Xanthoria* sp.). However, the corresponding E-values indicate that these similarities are statistically significant. Additional homology searches using the identified *Xanthoria* sp. sequences revealed a high percentage identity among homologs from other *Xanthoria* isolates (data not shown). Because the E-values support the identification of true homologs, no changes to the original interpretation were considered to be necessary.

Minor points:
Line 114-115: part of the sentence is missing

The sentence has been corrected

Line 119 "In summary …" I do not see in the previous sentences any indication that siderophore are involved in mutualistic interactions; the fact tha symbionts produce siderophore for their iron supply does not mean that it is involved in the symbiosis.

The sentence has been reworded to "In summary, siderophores are produced by fungal partners of mycorrhizal and endophytic associations and may play a role in symbiosis."

Table 1. Is the % cov for SitT is really of 131.2 % ? or is it a typo .

This is not a typographical error - it is the original readout from the NCBI blast search.

Suppl Table 3. A word is missing in the title of the table: "Homologous proteins involved or potentially involved in the microalgae Trebouxia sp., Chlamydomonas reinhardtii, Dunaliella and Phaeodactylum tricornutum »

Thank you. The legend title of Supplementary Table S3 (now Table S4) has been corrected.

Reviewer #4 (Remarks to the Author):

In Happacher et al., the authors explore, for the first time, the iron scavenging mechanisms of a lichen, Xanthoria parietina. The authors use a powerful combination of approaches, ranging from bioassays with Aspergillus fumigatus knockout mutants and X. parietina extracts, a BLAST-based genomic investigation of proteins in the putative X. parietina iron uptake and detoxification regulatory mechanisms, CESI-MS/MS identification of the putative ferrichrome produced by the X. parietina mycobiont, and finally some evidence of the mycobiont-derived ferrichrome's function in the X. parietina photobiont, Trebouxia decolorans.

Overall, the hypotheses are highly novel and well-investigated, and the work therefore warrants publication. The work is well-written and largely well-ordered and clear. The methodologies used are sound and state of the art, and very much cutting-edge when it comes to lichenological work. The methods section appear sufficiently detailed.

We are very grateful to the reviewer for the positive assessment and for the thoughtful and constructive feedback, which greatly contributed to improving the manuscript.

I have some minor suggestions and comments that I will outline below. My primary points of criticism/request for additional explanations or alternate interpretations have to do with the algal growth assay in Fig. 7 and its conclusions. Although the use of the mycobiont-derived siderophore by the alga to some degree appears evident, I am unconvinced so far by the specific interpretations made by the authors of the experimental result, and of the soundness of these specific data for Fig. 7 (i.e. the health of the algal cultures). However, this may be a difference in overall health of axenic cultures of T. decolorans relative to other Trebouxia species; if so, perhaps the authors can refer to additional literature demonstrating that these values are not abnormal for T. decolorans.

We thank the reviewer for raising this point regarding the physiological status of the algal cultures. The relatively low Fv/Fm values in Fig. 7 (~0.2–0.4) reflect the intentionally imposed iron stress rather than reduced viability. Iron limitation is known to constrain PSII efficiency, and the use of BPS in minimal medium was designed to accentuate this effect. Importantly, ferrichrome supplementation increased Fv/Fm from ~0.2 to ~0.4, indicating improved photosynthetic performance (values of ~0.4–0.6 are typical for healthy axenic cultures). This recovery demonstrates that the cultures were physiologically responsive and that the observed differences reflect relief of iron limitation. We therefore interpret Fv/Fm as a quantitative readout of iron stress, with its increase upon ferrichrome addition providing functional evidence that mycobiont-derived ferrichrome enhances iron availability to the photobiont under restrictive conditions.

Consistent with growth reduction under BPS-mediated iron limitation, algal growth increased upon a fivefold reduction in BPS concentration and was further enhanced when BPS was omitted entirely

- additional data have been added in Fig. 7. Under these conditions, the growth-promoting effects of both iron-free ferrichrome and the ferrichrome-iron complex are only marginal, likely due to uptake of iron via RIA in the absence of BPS.

L 99: “In RIA, ferric iron...” This should be the beginning of a new paragraph.

We have made this change as suggested.

LL 114-115: “Root tissues…” This sentence is missing a word (perhaps “contain”).

The sentence was corrected

LL 132-133: “Compared to…” Shouldn’t we expect that X. parietina, which often grows on relatively more heavy metal-polluted environments, produces less variable or makes less use of siderophores or generally engages in less iron scavenging than other lichen species that grow in iron-poor environments (e.g. many arctic or antarctic species)?

At the request of another reviewer, we included an *in silico* analysis of the conservation of iron homeostatic systems across a broad range of lichen mycobionts (Supplementary Table S3). This analysis revealed widespread conservation of both reductive iron assimilation and siderophore-mediated iron acquisition pathways among lichen mycobionts.

If this logic is deficient in the authors’ eyes, perhaps they could elaborate more on the rationale for using X. parietina for this initial study. Below the argument seems to be made that human-made habitats like concrete are iron poor, and that X. parietina’s ready colonization of these habitats implies a robust siderophore production for iron scavenging, but this should be stated clearly here.

With regard to our rationale, *X. parietina* was selected because it is a widely distributed lichen with genomic and transcriptomic resources available, thereby facilitating molecular interpretation. In addition, axenic cultures of both symbionts were available in the co-authors’ laboratories enabling production of sufficient biomass for biochemical and physiological analyses - an advantage that is very uncommon indeed among lichen mycobionts. We now added a brief statement in the Introduction to make this clear.

L 147: “…and potential for RIA and iron detoxification.” Mention of this could be made above in the rationale for using X. parietina. Additionally, mention of iron detoxification and its relationship to sideorphore production could be made more clearly in the earlier paragraphs.

We are grateful for this comment. To enhance clarity, we have reworded the sentence. The earlier paragraphs introduce iron homeostatic pathways characterized in fungi such as *A. fumigatus*, while the mentioned paragraph describes results. CccA-mediated iron detoxification has no direct link to siderophore production.

L 153: Maybe a line should be added here to describe explicitly what siderophores Sit2 is the expected transporter of, relative to Sit1 (my understanding was that both have overlapping transport ability and some specific transport ability).

The text has been reworded to ”The *ΔsidAΔftrAΔsit1AΔsit2* mutant is additionally defective in the Sit1 and Sit2 transporters, which are both responsible for uptake of coprogen-type siderophores and show partly overlapping as well as exclusive specificity for different ferrichrome-type siderophores. Thus, this mutant can utilize neither coprogen- nor ferrichrome-type siderophores with exception of a very limited growth response to ferricrocin.”

L 165/Fig. 1: “negativ control” -> negative control. I would also recommend the checks and Xs in panel B be made in different colors.

At the request of another reviewer, Fig. 1b has been removed.

L 240/Fig. 4: The algal side of this schematic seems misleadingly incomplete, even in its largely hypothetical state. Is there a reason the authors do not assume/do not explore the possibility that iron is ferried by larger transport forms after processing in the mycobiont cells to the photobiont?

We cannot exclude the possibility that, following processing in mycobiont cells, iron is transported to the photobiont in larger carrier forms; however, there is currently no experimental evidence supporting this scenario. In contrast, the data presented in Fig. 7 clearly demonstrate that *T. decolorans* can directly utilize the siderophore produced by the mycobiont without extracellular reduction of siderophore-bound iron. We therefore intentionally restricted our interpretation and discussion to this more parsimonious mechanism. We have revised the figure legend to make clear that the scheme is conceptual and does not aim to capture the full complexity of possible exchange pathways within the symbiosis.

L 297/Fig. 6: Maybe the authors should show some alternative genes/pathways not expected to be upregulated in concrete vs bark? What if this is just generally more biological activity in concrete relative to bark, sampling time, time of year, etc.?

The intention of this Fig. 6 was not to imply a global picture of transcriptional activity in mycobionts growing on concrete relative to bark, but rather to highlight substrate-associated, functionally coherent expression differences within a previously published dataset. Importantly, the differential expression analysis does not show a uniform increase in transcript abundance across the transcriptome in concrete-associated samples. Instead, genes implicated in iron acquisition and homeostasis, including *XpSidC*, are selectively up-regulated, whereas many other genes show no consistent difference between substrates (see Supplementary Table S1). This argues against a simple explanation based on overall higher biological activity, sampling time, or seasonal effects. Moreover, concrete represents an iron-poor and chemically distinct substrate compared to bark, providing an ecologically plausible context for the selective induction of iron acquisition pathways.

To further investigate potential substrate-dependent ferrichrome production by the *X. parientina* mycobiont, we analyzed ferichrome contents in three samples growing on bark and three on granite (new Fig. 6b). Ferrichrome content varied 4.7-fold, ranging from 1.8 pmol (1.3 ng) mg^{-1} dry mass in a bark sample to 8.5 pmol (6.3 ng) mg^{-1} dry mass in a granite sample. Although the mean ferrichrome content in granite samples was 1.8-fold higher than in bark samples, the difference was not statistically significant. Nevertheless, these results indicate that ferrichrome levels of *X. parietina* lichen thalli vary across substrates or sites, most likely reflecting differences in iron bioavailability. These results have been included in the main text. The Discussion has been revised to frame siderophore-related transcriptional changes and siderophore production as indicative of substrate-associated physiological adjustment rather than as direct evidence for organic versus organic substrates.

L 330/Fig. 7: Even though Fv/Fm values for lichen-forming algae are generally lower than normal for plants, these values all seem particularly very low. Even the cells receiving the ferrichrome-iron chelate are barely above 0.3, half of the values we usually see in lichen-forming Trebouxia cultures in the lab. Are the authors really sure that these cells were comparable/healthy enough to draw reasonable conclusions from? Or are these values to be expected from T. decolorans?

We appreciate the reviewer´s attention to the physiological status of the algal cultures. We agree that the absolute Fv/Fm values under the most restrictive conditions are low compared to those of healthy axenic cultures (~0.4–0.6). However, Fig. 7a shows that these values reflect severe but reversible iron stress (by siderophore supplementation) rather than loss of viability. Under 100 µM BPS, *T. decolorans* exhibits reduced growth and low Fv/Fm without ferrichrome or with $FeSO_4$

alone, whereas ferrichrome supplementation—especially as the iron complex—leads to progressive recovery of greening, biomass, and photosynthetic efficiency (Fig. 7a,b). When iron limitation is alleviated (lower or no BPS; new data in Fig. 7), all treatments display robust growth. We therefore interpret the low Fv/Fm under high-BPS conditions as a stress readout rather than evidence of compromised cultures. The consistent recovery upon ferrichrome addition confirms culture viability and supports the mechanistic conclusions. Corresponding data have been added to Fig. 7a and the text has been revised accordingly.

In addition, there is no statistical test seemingly being applied here. It is hard to tell if there is any meaningful biological difference between the iron-free ferrichrome and the bound version of the ferrichrome, perhaps, again, because the cells are so sickly/unhealthy to begin with, or perhaps because the iron-scavenging phenotype is not as clearly beneficial as the authors are claiming?

We thank the reviewer for this comment – statistical analysis has been included in Fig. 7b.

LL 388-389: "…suggesting that ferrichrome-iron chelates are most likely utilized by internalization." I am not clear on the authors' argument for why the iron-free version of the ferrichrome causing essentially the same growth potential in Trebouxia as the ferrichrome-iron chelate is consistent with the idea that the Trebouxia is using the mycobiont ferrichrome for iron scavenging. If anything, this seems to imply that the ferrichrome is somehow required by the photobiont for growth (as they mention in the subsequent sentences), but that iron scavenging itself is not generally required for growth of the photobiont.

Our working hypothesis is that *T. decolorans* acquires iron via two routes. First, reductive iron assimilation (RIA), involving extracellular reduction, reoxidation, and uptake via permeases, is supported by the presence of FreA-, FetC-, and FtrA-like homologues (new Table 2). Second, iron may be acquired through direct uptake of siderophore-iron complexes, possibly mediated by homologues of *Phaeodactylum tricornutum* ISIP1 or *Dunaliella salina* transferrin-like protein TTF-1 (new Table 2). BPS inhibits RIA by chelating ferrous iron produced by metalloreductases, thereby preventing reoxidation and subsequent uptake, thereby blocking reoxidation and uptake. Growth in the presence of BPS thus serves to distinguish between these pathways. The ability of *T. decolorans* to grow with ferrichrome-iron complexes despite BPS treatment indicates that siderophore-bound iron can be utilized independently of RIA.

Iron free ferrichrome promoted algal growth to a similar extent as ferrichrome iron complexes, likely due to its higher iron-binding affinity compared to BPS. Even under chelator excess, ferrichrome can scavenge trace iron from the medium - including BPS-bound iron -forming complexes that are subsequently taken up. Consistently, growth increases with reduced or omitted BPS (new data), most likely by RIA, confirming that iron scavenging underlies photobiont growth. The text has been revised to clarify this.

Otherwise, presumably the iron-free version of the ferrichrome would be expected to cause severe growth defects relative to the ferrichrome-iron chelate. If the authors disagree with this logic, I would be happy to read their argument here more clearly.

Our explanation: Ferrichrome is a strong iron chelator scavenging iron, even in the presence of excess BPS. If *T. decolorans* would not be able to utilize the ferrichrome-iron complex, ferrichrome supplementation would starve *T. decolorans* for iron like BPS but we see the opposite. This has been clarified in the Discussion.

Point-by-point response (in blue)

Reviewer #2 (Remarks to the Author):

The authors have addressed most of the concerns raised during the review process. I believe the revisions have significantly strengthened the manuscript, and the data now better support the conclusions. I am pleased to recommend this article for publication after some minor issues.

Minor issues: The author added a phylogenetic tree of SidC (Fig. 3c). The figure could be improved by removing bootstrap values smaller than certain values, leaving highly supported ones. The font for BS values can be bigger such that it may be more readable. Is the tree mid-point rooting or outgroup-rooting. The ancestral branch looks incomplete.

We would like to thank the reviewer for the positive feedback and valuable suggestions. We have edited Fig. 3c as suggested: the bootstrap values smaller than 80 have been removed, and the font size of the remaining values has been increased to improve readability. The displayed tree is unrooted and, as anticipated by the Reviewer, the ancestral branch is incomplete - for clarity only the clade containing close SidC homologues is shown. To clarify this, we have included a new Supplementary Fig. (Fig. S2) in supplementary information showing the full tree and describing the selection criteria for retrieving homologous proteins. The legend of Fig. 3c and the Methods section have been adapted accordingly.
